# Mapping the pneumococcal disease landscape in India: a meta-analysis of literature from 2007 to 2024 on serotype prevalence and antimicrobial resistance patterns

**DOI:** 10.1186/s12879-026-14389-x

**Published:** 2026-11-03

**Authors:** Santosh Taur, Mukundraj Keny, Namrata Kulkarni

**Affiliations:** 1 Department of Medical Affairs, Pfizer Ltd, Mumbai, Maharashtra India; 2 Senior Medical Advisor, Department of Medical Affairs, Pfizer Ltd, Mumbai, Maharashtra India

**Keywords:** Pneumonia, Serotypes, Antibiotic resistance, Pneumococcal conjugate vaccine, Invasive pneumococcal disease, Nasopharyngeal carriage

## Abstract

**Background:**

Pneumococcal disease remains a major public health concern in India, with significant prevalence in both children and adults. Pneumococcal conjugate vaccine (PCV) 13 provides high serotype coverage in children and in adults; however, non-vaccine serotypes are emerging. Rising antimicrobial resistance and limited local serotype data hinder effective disease control and vaccine policy. This meta-analysis consolidates evidence on pneumococcal serotype distribution, antimicrobial resistance (AMR)/non-susceptibility trends, and PCV coverage in the Indian population across pediatric and adult age groups.

**Methods:**

A systematic review and meta-analysis were conducted on pneumococcal serotype distribution and AMR/non-susceptibility patterns using Indian publications from 2007 to 2024. Comprehensive searches were performed in PubMed, Google Scholar, and EMBASE. Data were analyzed using a random-effects model in R (version 4.4.1), with heterogeneity assessed via the I² test. Serotype distribution was further stratified into invasive pneumococcal disease (IPD), carriage, and community-acquired pneumonia (CAP) subgroups.

**Results:**

Twenty-six studies, comprising 4,253 *Streptococcus pneumoniae (S. pneumoniae)* isolates, were included with 24 studies from the pre-Universal Immunization program (UIP) period. Among pediatric IPD estimates supported by multiple studies, serotype 14 (14.15%), serotype 19F (11.39%), and serotype 6B (10.17%) were most frequent. Higher estimates for pediatric IPD serogroups 6 (25%), 18 (12.5%), and 19 (10%) were each derived from one 40-isolate study and were considered exploratory. In the adult IPD population, serotype 1 (11.69%), and serotypes 23F (6.84%), and 19F (6.71%) were the most common. Adult CAP estimates came from one 50-isolate study, in which serotype 19A (14%) and serotype 8 (10%) were most frequent. AMR/non-susceptibility was consistently highest for cotrimoxazole/trimethoprim-sulfamethoxazole across pediatric IPD (86.16%) and adult IPD (85.24%) populations as well as in adult CAP (74%). Penicillin non-susceptibility was estimated at 14.33% (95% CI: 5.22–33.72) in pediatric IPD, although considerable between-study heterogeneity was observed (I² = 97.15). Accordingly, this pooled estimate should be interpreted in the context of the substantial variability across the included studies.

**Conclusion:**

Among pediatric IPD estimates supported by multiple studies, serotype 14, serotype 19F, and serotype 6B were most frequent. In the adult IPD population, serotype 1, and serotypes 23F, and 19F were the most common. Cotrimoxazole/trimethoprim-sulfamethoxazole showed the highest resistance. Among PCV formulations assessed, PCV20 showed the highest serotype coverage. There is a need for continuous, geographically broader surveillance of serotypes and AMR patterns in pneumococcal disease to guide the national vaccination policies and choice of PCVs.

**Supplementary Information:**

The online version contains supplementary material available at https://doi.org/10.1186/s12879-026-14389-x.

## Introduction

The persistent bacterium *S. pneumoniae* has long been one of humankind’s greatest foes. First identified by Louis Pasteur and George Sternberg more than a century ago [1], pneumonia accounts for 20% of under-five deaths around the world [2]. A similar pattern has been observed in national survey data, which shows that 14% of child deaths were due to pneumonia in 2017, making it the second biggest killer of children under five in 2017 [3, 4]. A meta-analysis of 12 studies involving 1,435 patients found that around one-fifth of adult Indian patients with community-acquired pneumonia (CAP) were infected with *S. pneumoniae*, indicating that it is a predominant causative organism of CAP compared to other pathogens [5]. 

Pneumococci are spherical, Gram-positive bacteria whose virulence is primarily driven by their capsular polysaccharides, crucial for immune evasion and vaccine design [6]. Over 100 distinct serotypes, grouped into serogroups based on capsule structure, vary in prevalence and invasiveness. Although many exist, only a few cause the most invasive diseases [7, 8], with distribution influenced by region, age, and time [9, 10]. 

AMR worsens the burden of disease by prolonging hospital stays, increasing mortality, and driving up healthcare costs [11]. In India, a study of 86 *S. pneumoniae* isolates (2018–2021) showed a sharp decline in susceptibility to erythromycin (71.4% to 16.2%), meropenem (85.7% to 35.1%), and penicillin (37.5% to 27%) [12]. AMR also imposes significant economic costs, increasing direct medical expenses by 4% and total costs by 5%, mainly due to hospitalizations from drug-resistant infections [13]. The rise of resistant strains in the community highlights the need for ongoing surveillance and broader serotype coverage [14]. 

Prevention of pneumococcal disease primarily relies on vaccination, a key strategy for reducing disease burden in both children and adults, while prophylactic antibiotics are used only in specific cases [15]. Vaccination promotes herd protection by lowering transmission [1]. Since the first use of purified capsular polysaccharide in 1937, vaccine development has advanced significantly [16]. The seven-valent conjugate vaccine (PCV7), licensed in 2000 [17, 18], was effective not only against disease but also in preventing nasopharyngeal colonization, indirectly protecting unvaccinated individuals [19]. 

To address serotype replacement [20], broader-spectrum vaccines, PCV10-Protein D, TT, & DT (including 4, 18C) and PCV13, were introduced in 2010 [21], with PCV13 significantly reducing disease burden and antibiotic use in children and adults. Newer 15- and 20-valent vaccines (PCV15 and PCV20), approved in the U.S. in 2022, further extend coverage.

The Community-acquired Pneumonia Immunization Trial in Adults (CAPITA) showed PCV13 reduced vaccine-type CAP by 46% and IPD by 75% in adults aged > 65 years [22, 23]. Interrupted time-series analysis showed that PCV13 paediatric vaccination programs significantly reduced outpatient antibiotic use in young children [24]. Unlike broad-spectrum antibiotics, vaccines prevent disease and reduce antibiotic dependence [25]. 

Expanding serotype coverage is vital for further reducing invasive pneumococcal disease (IPD) and antibiotic reliance. However, vaccine effectiveness depends on local serotype distribution, making accurate, region-specific surveillance essential [26]. In India, low pediatric vaccine uptake is unlikely to produce herd protection, in unvaccinated groups such as older adults. Moreover, the absence of robust, representative serotype data due to limited lab infrastructure hampers vaccine policy development [27, 28]. 

This systematic review and meta-analysis aim to assess pneumococcal serotype distribution across age groups in India, examine AMR patterns, and evaluate current vaccines serotype coverage. The findings can support evidence-based vaccination strategies tailored to the Indian context.

## Methods

We followed PRISMA guidelines and registered the protocol for this systematic review and meta-analysis with the PROSPERO systematic protocol registry (https://www.crd.york.ac.uk/PROSPERO/view/CRD42024559520).

We performed a systematic literature review of the published literature from January 2007 to May 2024 to identify articles that describe the pneumococcal serotypes in children and adults in India. The introduction of PCV7 in the early 2000s marked a global shift in pneumococcal serotype distribution and antimicrobial resistance [29]. Trends from 2007 to 2024 reflect changes driven by vaccination and antimicrobial use, underscoring the need for ongoing surveillance to guide current and future pneumococcal control efforts. Therefore, we considered this time frame for our review.

### Literature search strategy

We searched the following literature databases: PubMed, Embase, and Google Scholar, and considered studies for the review that were published in the English language. Search included keywords and medical subject headings (MeSH) terms related to “Streptococcus pneumoniae” “serotype distribution” “antimicrobial resistance” “PCV” and “India”. Conference abstracts from the International Conference of Infectious Diseases (2020) were accessed for information. The search was further supplemented by the reviews of reference lists and bibliographies. Where published articles did not provide complete serotype or AMR data, we contacted the corresponding authors to request missing, or supplemental data. Two reviewers independently selected articles meeting the inclusion criteria based on title, abstract, or keywords. Discrepancies were resolved by consensus. In case of remaining dissenting views, the third reviewer was consulted. Subsequently, one reviewer read and analyzed selected studies for eligibility. Citations and reference lists from review articles found in the initial search were checked to ensure that no studies were missed.

### Inclusion and exclusion criteria

We included published observational studies conducted in the Indian pediatric and adult population and any study design (prospective or retrospective) that reported data on different *S. pneumoniae* serotypes. The samples were collected from either normally sterile sites (e.g. blood, cerebrospinal fluid, pleural fluid) or nasopharyngeal swabs/aspirates or throat swabs or pharyngeal swabs and assessed either through culture or molecular methods. We selected articles where the Quellung reaction alone or in combination with other serotyping methods was used. However, we excluded those studies that had both the population (pediatric and adult) without split and those that provided data only on the antibiotic resistance. We also excluded pneumococcal surveillance studies published before 2007, systematic reviews, abstracts without full-text, studies written in languages other than English case reports, commentaries or letter to editors, studies in animals, studies reporting vaccine serotype coverage for selected strains, and vaccine trials.

### Outcomes

Primary outcome: Distribution of *S. pneumoniae* serotypes among the diseased and carriages in pediatric and adult age groups in India.

Secondary outcome: (a) Prevalence of antimicrobial resistance among the *S. pneumoniae* isolates. (b) Proportion of pneumococcal serotypes present in the globally and/or locally available PCVs.

### Quality assessment

Study limitations and risk of bias were assessed using the Newcastle-Ottawa Scale (NOS) for assessing the quality of nonrandomised studies in meta-analyses [30]. Although the original NOS framework evaluates study quality across selection, comparability, and outcome/exposure domains with a maximum score of 9, the scale was modified because the included studies were heterogeneous in design, comprising prospective and retrospective hospital-based studies, surveillance studies, carriage studies, cross-sectional studies, and laboratory-based studies reporting pneumococcal serotype distribution and/or antimicrobial resistance. As several items in the original NOS are tailored to conventional cohort or case-control studies and were not uniformly applicable across these study designs, a modified NOS was used. The modified NOS assessed five domains relevant to the included studies: representativeness of the study population (selection bias), sample size adequacy (selection bias), exposure/serotyping ascertainment (selection bias), comparability of study groups (comparability bias), and appropriateness of statistical analysis (outcome bias). Each domain was scored 0 or 1, yielding a maximum score of 5.

Each study was assessed for risk of bias independently by two reviewers. Conference abstract and author-provided supplemental data were assessed using the same modified NOS-based risk-of-bias procedure as published studies, where sufficient information was available. A third reviewer was consulted when there was disagreement between the two independent reviewers regarding the quality assessment. Studies with scores ≥ 4 (out of 5) were deemed high quality/low risk of bias, scores of 3 and 2 (out of 5) were deemed medium quality/some risk of bias, and score ≤ 1 were deemed low quality/high risk of bias.

### Data collection, extraction and management

Structured model tables developed in Microsoft Excel spreadsheet were used to extract and organize data on the following variables: authors, study title, year of publication, patient population (pediatric or adult), study reporting serological data only or with antimicrobial resistance data, Indian city of study, study design, duration of study, age range of the studied cohort, type of pneumococcal disease (invasive or non-invasive [like CAP]) or carriage, number of patients, number of isolates, mode of specimen collection, mode of specimen analysis, number of isolates positive for the specific pneumococcal serotypes, and proportion of pneumococcal isolates resistance for the antibiotics, such as penicillin, Ceftriaxone, Fluoroquinolone/Levofloxacin, chloramphenicol, cotrimoxazole/trimethoprim-sulfamethoxazole, erythromycin and tetracycline. Although two studies included in our meta-analysis reported antimicrobial resistance using the term “trimethoprim-sulfamethoxazole,” while others used “cotrimoxazole,” these terms refer to the same antibiotic combination. Similarly, only one study reported separate AMR data for fluoroquinolones, while 3 reported AMR data for levofloxacin. To avoid duplicate interpretation these were treated as the same antibiotic combination and analyzed as cotrimoxazole/trimethoprim-sulfamethoxazole and fluoroquinolones/levofloxacin. The data were double-entered and underwent several data checks to ensure completeness and accuracy of the extracted data. For any discrepancies, the final consensus was made after discussion with the third reviewer.

### Analysis

As detailed in Table 1, each reported serotype was categorized according to whether it was included in currently available pneumococcal vaccine formulations. Serotypes were grouped under the specific vaccine categories - PCV10-Protein D, TT, & DT (including 4, 18C), PCV10-CRM_197_ (including 6A, 19A), PCV13, PCV14, PCV15 (including 2, 12F), PCV15 (including 22F, 33F), PCV20, PCV21, and PPSV23. In addition, serotypes that could not be clearly identified were placed in the undifferentiated category, which included both non-typeable isolates (where serotyping was attempted but inconclusive) and non-classified isolates (where results were reported as grouped serotypes, e.g., 6A/B/C or 15B/C). Isolates not included in PCV20 and the undifferentiated category were grouped as non-PCV20 serotypes, representing all remaining clearly identifiable serotypes outside the PCV20 formulation.

**Table 1:** Definitions for pneumococcal vaccine formulation serotype coverage estimates

PCV Formulation Serotypes	Definition
PCV10-Protein D, TT, & DT (including 4, 18C) [GlaxoSmithKline **(**GSK)]	1, 4, 5, 6B, 7F, 9V, 14, 18C, 19F, 23F
PCV10-CRM_197_ (including 6A, 19A) [Serum Institute of India Pvt. Ltd. (SII)]	1, 5, 6A, 6B, 7F, 9V, 14, 19A, 19F, 23F
PCV13 (Pfizer)	1, 3, 4, 5, 6A, 6B, 7F, 9V, 14, 18C, 19A, 19F, 23F
PCV14 [Biological E. Limited (BE)]	1, 3, 4, 5, 6B, 7F, 9V, 14, 18C, 19A, 19F, 22F, 23F, 33F
PCV15 (including 2, 12F) [Aurobindo / Tergene Biotech (Auro/Tr)]	1, 2, 3, 4, 5, 6A, 6B, 7F, 9V, 12F, 14, 18C, 19A, 19F, 23F
PCV15 (22F, 33F) (Merck)	1, 3, 4, 5, 6A, 6B, 7F, 9V, 14, 18C, 19A, 19F, 22F, 23F, 33F
PCV20 (Pfizer)	1, 3, 4, 5, 6A, 6B, 7F, 8, 9V, 10A, 11A, 12F, 14, 15B, 18C, 19A, 19F, 22F, 23F, 33F
Non PCV20 serotypes	Serotypes that are not included in PCV20
PCV21 (Merck & Co.)	3, 6A, 7F, 8, 9N, 10A, 11A, 12F, 15A, 15C, 16F, 17F, 19A, 20A, 22F, 23A, 23B, 24F, 31, 33F, 35B
PPSV23 (Merck & Co.)	1, 2, 3, 4, 5, 6B, 7F, 8, 9N, 9V, 10A, 11A, 12F, 14, 15B, 17F, 18C, 19A, 19F, 20, 22F, 23F, 33F
**Other Serotype Categories**	
Undifferentiated	Serotype results that do not allow one to distinguish between any of the available PCV vaccines. This includes results where serotypes are described as “non-typeable” and “non-classified”. • **Non-typeable**: Serotyping was attempted, but the specific serotype could not be identified. • **Non-classified**: Results that are reported as a group of closely related serotypes (e.g., 6A/B/C, 9A/L, 10C/F, 15B/C). These are assigned to their corresponding *serogroup* (e.g., 6A/B/C is classified under serogroup 6).

(TT- Tetanus Toxoid; DT- Diphtheria Toxoid; CRM-Cross Reactive Material 197)

### Meta-analysis

Preliminary analysis of the abstracted data was performed on the Microsoft Excel spreadsheet program. The data were analyzed to determine the percentage distribution of pneumococcal serotypes which was calculated by dividing the number of isolates for each serotype with the total number of isolates included in all the studies. We conducted meta-analyses for serotypes and antimicrobial resistance using RevMan (version 5.3; Nordic Cochrane Centre) and R (version 4.4.1).

For prevalence pooling, individual study proportions were first transformed using the logit transformation (PLOGIT), then random effects models were used to pool estimates by stratum. The pooled estimates were then back-transformed and presented as percentages with 95% confidence intervals. When data were available, we performed sub-analyses based on different pediatric age groups. A predominant serotype was defined as the serotype that had a distribution percentage > 5% of the total isolates [31]. The prevalence of the antimicrobial resistance was defined as the percentage of isolates that were reported as resistant, intermediate resistant, or non-susceptible out of the total number of isolates tested for the specific antimicrobial agent [32]. Therefore, the pooled estimates represent antimicrobial resistance/non-susceptibility rather than complete resistance alone. Study heterogeneity was assessed using the I² statistic for all pooled serotype-specific and antimicrobial-resistance meta-analyses. I² values were interpreted as low, moderate, and high heterogeneity at approximately 25%, 50%, and 75%, respectively. For serotypes or antibiotics represented by only one study within a subgroup, heterogeneity statistics were reported as not applicable (NA). Between-study variance (Tau²) was estimated using the Restricted Maximum-Likelihood (REML) method. Individual studies were weighted using the inverse-variance approach within the random-effects model. Because separate meta-analyses were performed for each serotype and serogroup, study weights varied across analyses according to the corresponding prevalence estimate, within-study variance, and between-study variance (τ²). The potential vaccine serotype coverage was determined by calculating the sum of the vaccine-type serotype proportion in the respective vaccines and presented as percentages. Because the definition of a predominant serotype (> 5% of isolates) does not account for the number of contributing studies, estimates derived from a single study were flagged as single-study observations and interpreted as exploratory rather than as pooled estimates, and the ranking of the most prevalent types emphasizes estimates supported by multiple (≥ 3) studies. In addition, where I² exceeded 75%, pooled estimates were considered to have very high between-study heterogeneity and were interpreted as approximate summaries, emphasizing the range of study-level estimates rather than the point estimate.

## Results

### Data review

**Fig. 1:**
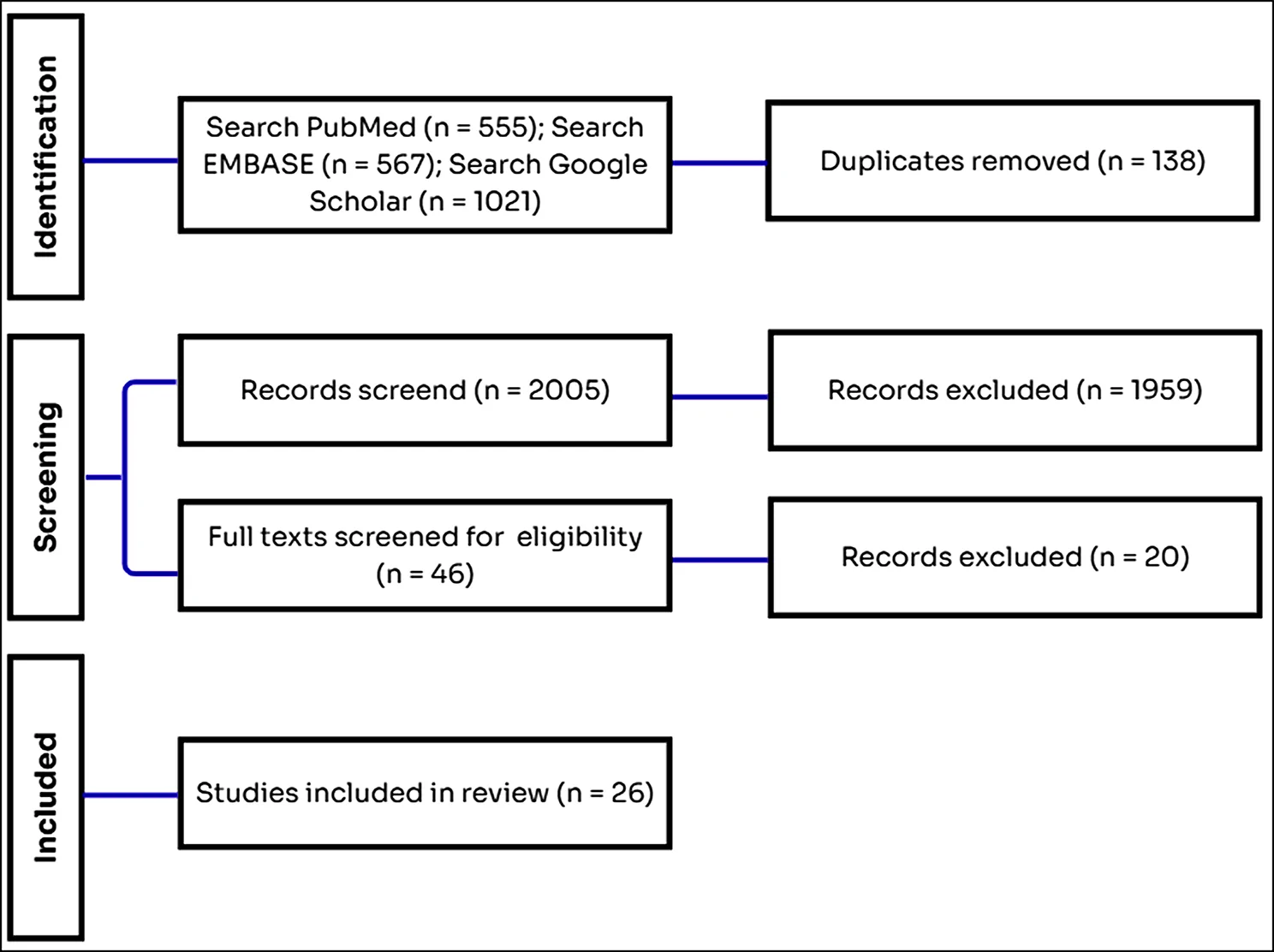
PRISMA-2020 flow diagram illustrating the selection process of studies

We screened a total of 2005 articles and abstracts in our preliminary search, of which 46 studies fulfilled inclusion criteria. Among them 20 studies were further excluded: 8 included combined pediatric and adult populations without separation, and 12 reported data solely on the antibiotic resistance. After selection, 26 studies were finally included and analyzed (Fig. 1), of which 22 reported both serological and AMR data, while 4 reported data only on the surveillance of pneumococcal serotypes.

### Risk of bias assessment

A methodological quality assessment of the included studies was performed using three key domains of bias: selection, comparability, and outcome. Most studies demonstrated a low risk of bias in terms of the representativeness of the study population and the ascertainment of exposure, indicating appropriate sampling and reliable identification of *S. pneumoniae* serotypes. However, sample size emerged as a notable concern. (Add. File 2-Supp. Figure 1)

Comparability of subjects across outcome groups showed mixed results, with a moderate number of studies lacking adequate adjustment for confounding variables, contributing to both high and unclear risk of bias. Similarly, while most studies used appropriate statistical methods, a few were marked by high risk due to suboptimal or inadequately reported analyses.

Overall, most included studies were methodologically sound in exposure assessment and representativeness, except for ‘statistical outcome bias,’ which showed a low-to-medium risk of bias across the included studies.

### Study characteristics

Key characteristics of the included studies are summarized in Add. File 1-Table 1. Among the 26 studies reviewed, the majority were conducted in hospital settings, either at a single center or across multiple institutions. Most of these were designed as prospective investigations, while 4 followed a retrospective approach. Surveillance studies were also notably represented, with 10 studies forming part of multi-center efforts, national surveillance programs, or international collaborations. Additionally, approximately 8 studies were primarily laboratory-based, focusing on microbiological or molecular analyses. Included studies were geographically unevenly distributed, with a greater contribution from tertiary-care and surveillance sites in Southern India (20), followed by Northern India (11), while Eastern and Western India were represented in only 3 studies each. Multicentric studies were counted once for each region represented. Add. File 1-Table 1.

Clinical samples varied by study type and included cerebrospinal fluid (CSF), pleural fluid, blood, tracheal aspirates, nasopharyngeal swabs, and sputum. For non-culture identification of *S. pneumoniae*, blood, CSF, and pleural fluid specimens were tested using polymerase chain reaction (PCR). Serotyping methods included the Quellung reaction, coagglutination, conventional culture, and sequential multiplex PCR.

AMR was assessed using E-tests and agar dilution to determine minimum inhibitory concentrations (MICs), following Clinical and Laboratory Standards Institute (CLSI) guidelines [33]. According to the CDC, Multidrug resistance (MDR) is defined as microorganisms, predominantly bacteria, that are resistant to one or more classes of antimicrobial agents [34]. In culture-negative but PCR-positive samples, molecular typing was conducted by amplifying the capsular polysaccharide synthesis (CPS) region, followed by Sanger sequencing of the amplicons. Colonization in children was confirmed through culture or PCR detection of *S. pneumoniae* [35]. 

### Meta-analysis of *Streptococcus pneumoniae* serotypes

This meta-analysis synthesized data from 26 eligible studies encompassing 4,253 isolates of *S. pneumoniae* collected across various regions in India with 24 studies from the pre-Universal Immunization program (UIP) period. A total of 102 distinct serotypes were identified, providing a comprehensive overview of serotype distribution across age groups and clinical presentations. Pooled effect sizes reinforced the dominance of these serotypes. Heterogeneity varied across serotype-specific analyses and is reported for each pooled estimate using I² and Tau² in the corresponding tables and forest plots. For analyses based on a single study, heterogeneity was reported as not applicable.

### Pediatric analysis

**Fig. 2:**
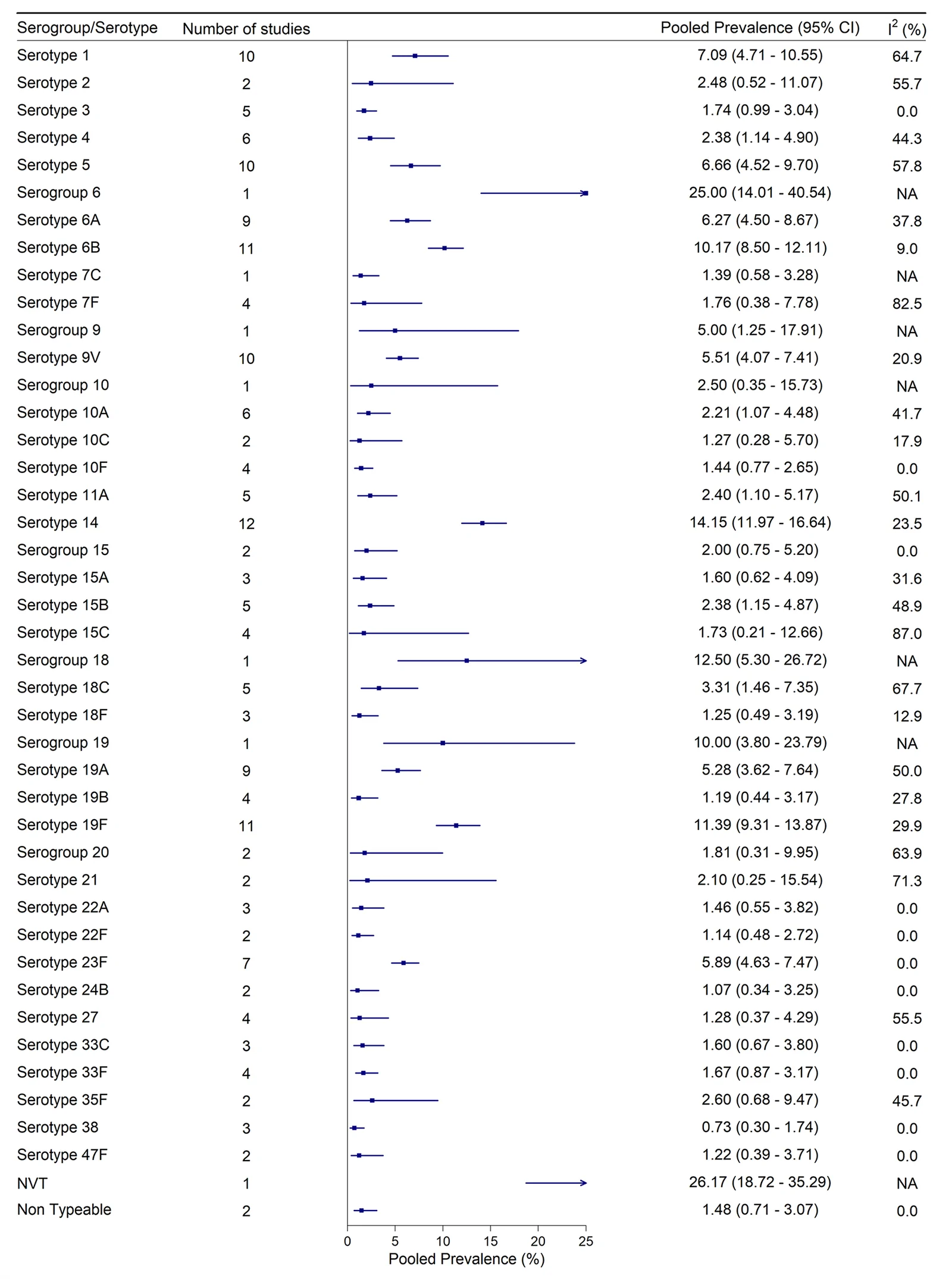
Forest plot showing pooled prevalence estimates across the pediatric IPD population with 95% confidence intervals and heterogeneity statistics, including I² and Tau², where estimable

**Fig. 3:**
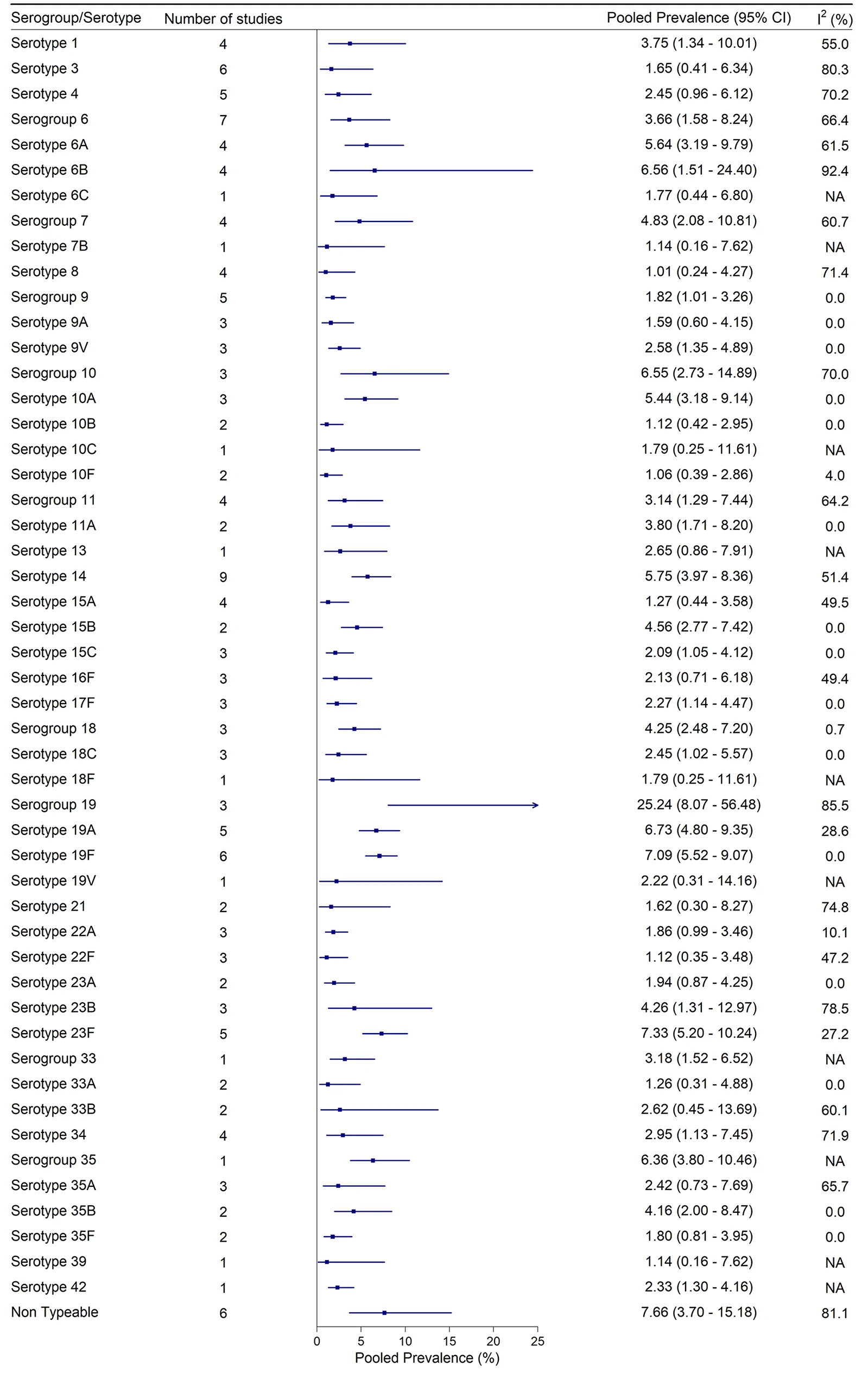
Forest plot showing pooled prevalence estimates across the pediatric carriage population with 95% confidence intervals and heterogeneity statistics, including I² and Tau², where estimable

In the pediatric IPD population, serogroup 6 had the highest effect size at 25% (95% CI 14.01–40.54), followed by serotype 14 at 14.15% (95% CI 11.97–16.64), serogroup 18 at 12.5% (95% CI 5.30-26.72), serotype 19F at 11.39% (95% CI 9.31–13.87), serotype 6B at 10.17% (95% CI 8.50-12.11), serogroup 19 at 10% (95% CI 3.80-23.79), serotypes 1 at 7.09% (95% CI 4.71–10.55), 5 at 6.66% (95% CI 4.52–9.70), serotypes 6A at 6.27% (95% CI 4.50–8.67), 23F at 5.89% (95% CI 4.63–7.47), 9V at 5.51% (95% CI 4.07–7.41), and 19A at 5.28% (95% CI 3.62–7.64). (Table 2; Fig. 2, Add. File 2-Supp. Table 1) In the pediatric carriage population, serogroup 19 was the most prevalent, comprising 25.24% (95% CI 8.07–56.48), followed by serogroups 10 at 6.55% (95% CI 2.73–14.89), 35 at 6.36% (95% CI 3.80-10.46), and serotype 14 at 5.75% (95% CI 3.97–8.36). Among serotypes, 23F was most common, accounting for 7.33% (95% CI 5.20-10.24), followed by 19F at 7.09% (95% CI 5.52–9.07), 19A at 6.73% (95% CI 4.80–9.35), 6B at 6.56% (95% CI 1.51–24.40), 6A at 5.64% (95% CI 3.19–9.79), and 10A at 5.44% (95% CI 3.18–9.14). (Table 3; Fig. 3, and Add. File 2-Supp. Table 2) Notably, the pediatric IPD estimates for serogroups 6, 18, and 19 (25%, 12.5%, and 10%, respectively) were each derived from a single 40-isolate study (k = 1) and therefore represent single-study observations rather than pooled estimates; their confidence intervals reflect within-study sampling variability only, and between-study heterogeneity was not estimable. These figures should be interpreted with caution relative to the multi-study estimates for serotypes 14, 19F, and 6B.

**Table 2:** Meta-analysis results of the most common serogroups/serotypes in the pediatric IPD population

Serogroups/ Serotypes	Total No. of Isolates	Number of studies (k)	Pooled Prevalence (%)	95% CI	Tau²	I²
Serogroup 6	40	1	25.00	14.01–40.54	NA	NA
Serotype 14	1341	12	14.15	11.97–16.64	0.0252	23.5
Serogroup 18	40	1	12.50	5.30-26.72	NA	NA
Serotype 19F	1147	11	11.39	9.31–13.87	0.0401	29.9
Serotype 6B	1301	11	10.17	8.50-12.11	0.01	9
Serogroup 19	40	1	10.00	3.80-23.79	NA	NA
Serotype 1	1163	10	7.09	4.71–10.55	0.2683	64.7
Serotype 5	1189	10	6.66	4.52–9.70	0.2243	57.8
Serotype 6A	1080	9	6.27	4.50–8.67	0.1011	37.8
Serotype 23F	1119	7	5.89	4.63–7.47	0	0
Serotype 9V	1163	10	5.51	4.07–7.41	0.0516	20.9
Serotype 19A	1263	9	5.28	3.62–7.64	0.1574	50

Common serogroups/serotypes (≥ 5% representation) are shown here

**Table 3:** Meta-analysis results of the most common serogroups/serotypes in the pediatric carriage population

Serogroups/ Serotypes	Total No. of Isolates	Number of studies (k)	Pooled Prevalence (%)	95% CI	Tau²	I²
Serogroup 19	119	3	25.24	8.07–56.48	1.2012	85.5
Serotype 23F	721	5	7.33	5.20-10.24	0.0467	27.2
Serotype 19F	834	6	7.09	5.52–9.07	0	0
Serotype 19A	789	5	6.73	4.80–9.35	0.0466	28.6
Serotype 6B	526	4	6.56	1.51–24.40	2.0597	92.4
Serogroup 10	326	3	6.55	2.73–14.89	0.4509	70
Serogroup 35	220	1	6.36	3.80-10.46	NA	NA
Serotype 14	1312	9	5.75	3.97–8.36	0.1671	51.4
Serotype 6A	701	4	5.64	3.19–9.79	0.2206	61.5
Serotype 10A	257	3	5.44	3.18–9.14	0	0

### Segregated pediatric assessment

**Fig. 4:**
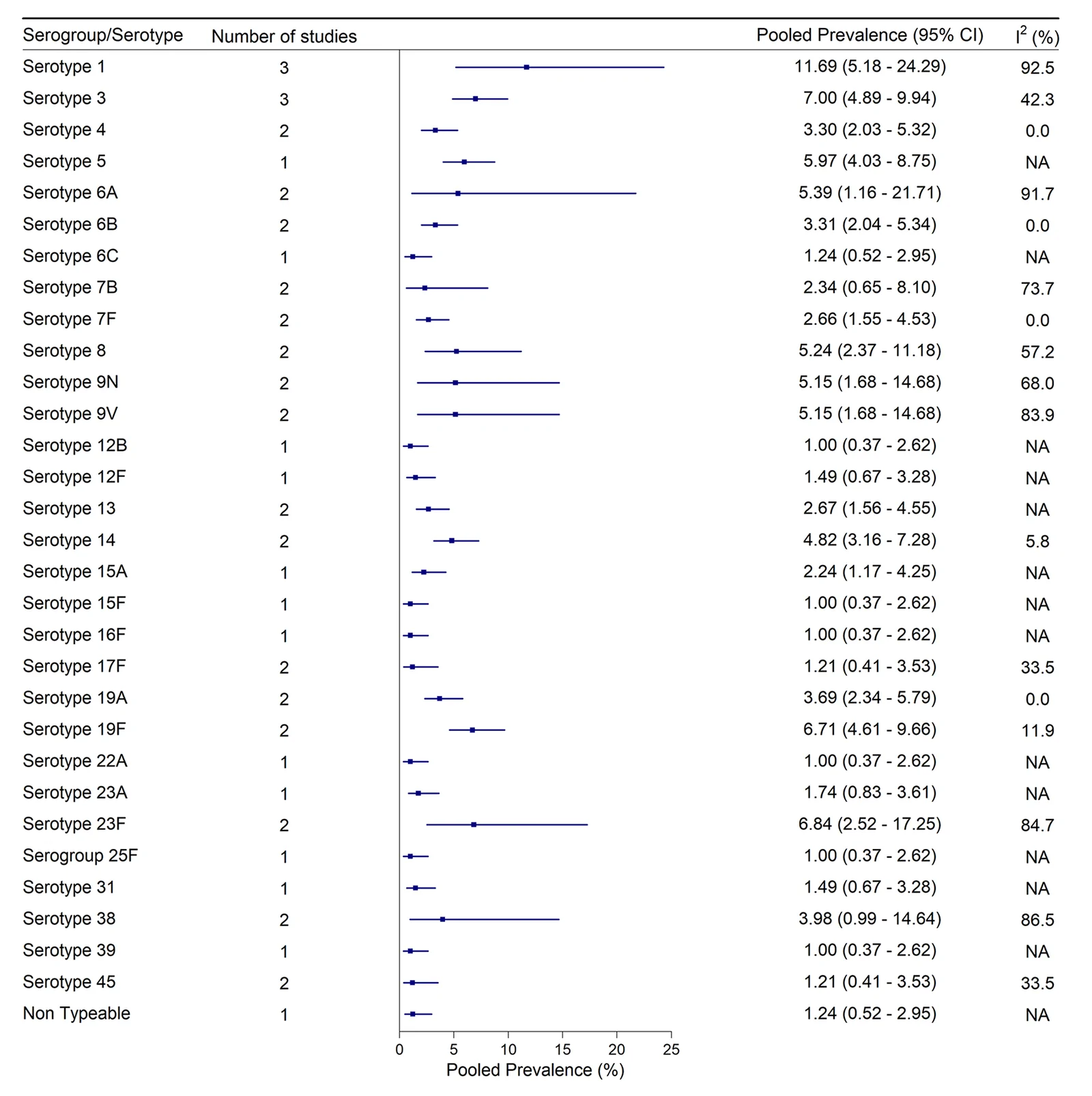
Forest plot showing pooled prevalence estimates across the adult IPD population with 95% confidence intervals and heterogeneity statistics, including I² and Tau², where estimable

**Fig. 5:**
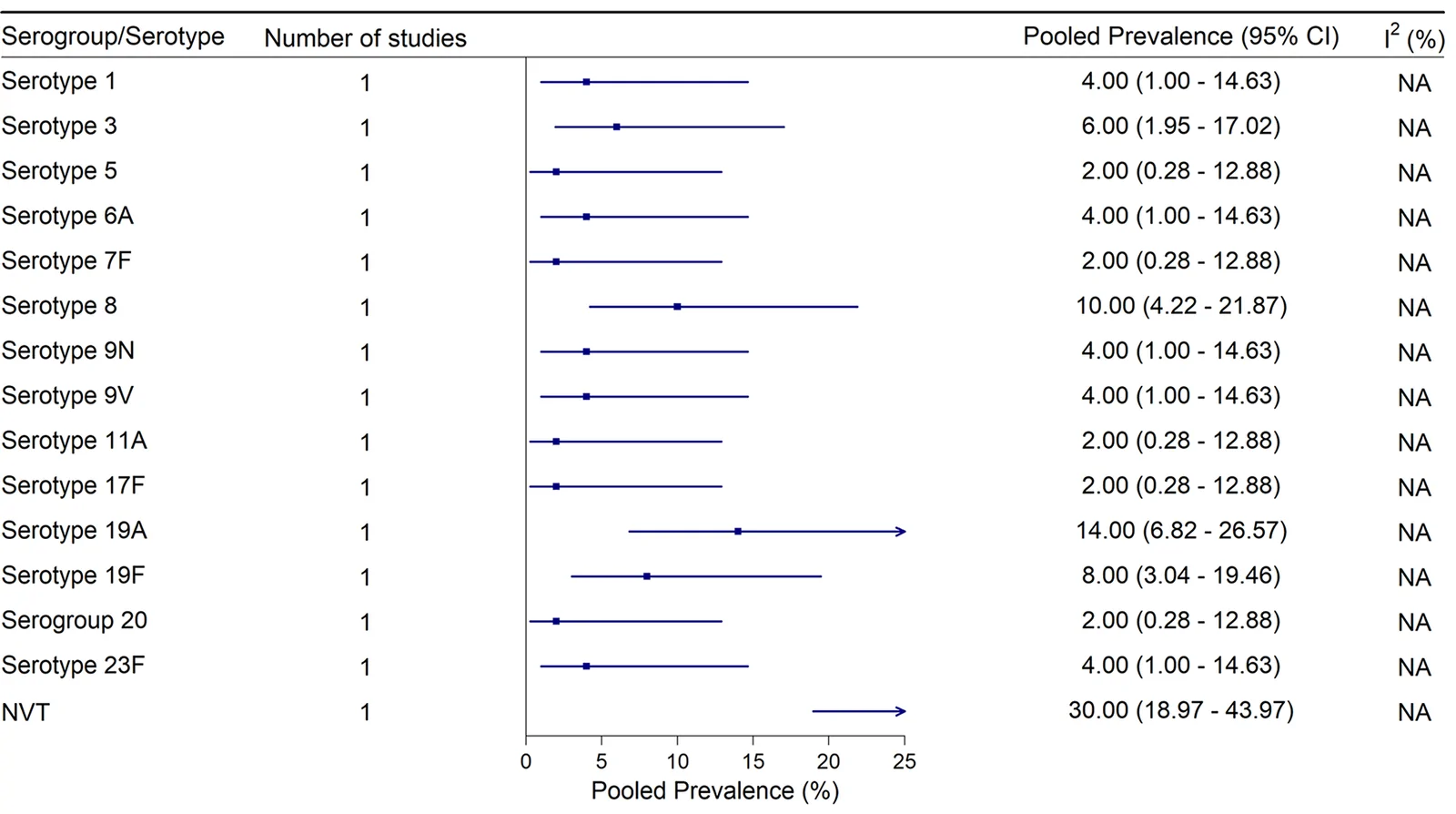
Forest plot showing pooled prevalence estimates across the adult CAP population with 95% confidence intervals and heterogeneity statistics, including I² and Tau², where estimable

In children under two years of age, serogroup 6 was particularly dominant, comprising 28.6% of isolates, while 23F (22.22%) and 19F (16.24%) were the most frequent serotypes. In the 3–5-year age group, 19A (12.46%) and 23F (11.9%) were most common, while older children and adolescents (5–24 years) showed higher representation of serogroup 4 (12.26%) and serotypes 6A (12.7%) and 15B (11.11%). (Table 4, Add. File 2-Supp. Tables 3, 4, 5 and 6, Supp**.** Figures 2–5**)**

**Table 4:** Proportion of the most common serogroups/serotypes in the different pediatric age groups

*≤* 2 years 3 studies^a^ 599 isolates		3–5 years 1 study^b^ 90 isolates		5–24 years 3 studies^c^ 131 isolates		*≤* 5 years 18 studies^d^ 2312 isolates	
6 (28.57%)	23F (22.22%)	5 (12.5%)	19A (12.46%)	4 (12.26%)	6A (12.7%)	19 (25.4%)	19F (9.61%)
14 (8.05%)	19F (16.24%)	1 (8.33%)	23F (11.9%)	20 (5.56%)	15B (11.11%)	14 (10.88%)	6B (8.47%)
19 (10.7%)	6A (11.66%)	15 (6.25%)	19F (10%)	13 (5.56%)	19F (9.45%)	10 (9.56%)	6A (7.17%)
9 (7.14%)	11A (11.11%)		18C (9.5%)	8 (5.36%)	6B (5.88%)	7 (8.9%)	23F (6.69%)
18 (7.14%)	18C (11.11%)		6B (7.19%)	38 (5.56%)	19A (5.88%)	1 (7.71%)	19A (5.77%)
1 (8.01%)	6B (8.89%)		6A (7.14%)		15A (5.56%)	5 (5.88%)	
5 (5.98%)					22F (5.56%)	18 (5.97%)	
					35A (5.36%)		
					33B (5.36%)		
					16F (5.36%)		

**a**: 1 study (Ravi Kumar KL et al., 2013 [36])- age group 28 days to 20 months; 1 study (Rupa V et al., 2014 [37]) – age group 0 to 2 years, and Nagaraj G et al., 2021 [38])- age group ≤ 2 years

**b**: 1 study (Nagaraj G et al., 2021 [38])- age group 3–5 years

**c**: 1 study (Nagaraj G et al., 2021 [38])- age group 5–24 years; 1 study (Verghese VP et al., 2017 [11])- age group 5–16 years; 1 study (Samson D et al., 2020 [39])- Cohort followed and reported at 8th year of life

**d**: 7 studies (Verghese VP et al., 2017 [11]; Gopi T et al., 2016 [40]; John J et al., 2018 [41]; Manoharan A et al., 2017 [42]; Nagaraj S et al., 2017 [43]; Rafiqullah IM et al., 2024 [44]; Varghese R 2021 [45]) – age group less than 6 years; 3 studies (Sutcliffe CG et al. 2019 [46]; Gupta P et al., 2022 [47]; Verma N et al., 2023 [48]) – age group 2 to 59 months; 1 study (Wattal C et al., 2007 [49]) – age group 3 months to 3 years; 1 study (Vandana G et al., 2016 [50]) – age group 6 weeks to 5 years; 1 study (Kumar G et al., 2021 [51]) - age group 1 month to 59 months; 2 study (Kumar KL et al., 2014 [52], Nandan TM et al., 2013 [53]) - age group 3 months to 5 years; 1 study (Raman R et al., 2017 [54]) - age group 6 months to 5 years; 1 study (Nisarga et al., 2015 [55]) - age group 28 days to < 5 years; 1 study (Balaji V et al., 2015 [56]) - age group 2 months to 5 years

### Adult analysis

In the adult IPD population, serotype 1 was most prevalent, accounting for 11.69% (95% CI 5.18–24.29) of isolates, followed by serotype 3 at 7% (95% CI 4.89–9.94), serotype 23F at 6.84% (95% CI 2.52–17.25), serotype 19F at 6.71% (95% CI 4.61–9.66), serotype 5 at 5.97% (95% CI 4.03–8.75), serotype 6A at 5.39% (95% CI 1.16–21.71), serotype 8 at 5.24% (95% CI 2.37–11.18), serotype 9N at 5.15% (95% CI 1.68–14.68) and serotype 9V at 5.15% (95% CI 1.68–14.68) further supporting the importance of these serogroups/serotypes in IPD among older individuals. (Table 5; Fig. 4, and Add. File 2-Supp. Table 7)

Among the adult CAP population, serotype 19A was the most common, accounting for 14% (95% CI 6.82–26.57), followed by serotype 8 at 10% (95% CI 4.22–21.87), serotype 19F at 8% (95% CI 3.04–19.46), and serotype 3 at 6% (95% CI 1.95–17.02). (Table 6; Fig. 5, and Add. File 2 Supp. Table 8) All adult CAP estimates were derived from a single study (k = 1) and are exploratory; between-study heterogeneity was not estimable, and the confidence intervals reflect within-study sampling variability only.

**Table 5:** Meta-analysis results of the most common serogroups/serotypes in the adult IPD population

Serogroups/Serotypes	Total No. of Isolates	Number of studies (k)	Pooled Prevalence (%)	95% CI	Tau²	I²
Serotype 1	906	3	11.69	5.18–24.29	0.4552	92.5%
Serotype 3	906	3	7.00	4.89–9.94	0.0467	42.3%
Serotype 23F	490	2	6.84	2.52–17.25	0.481	84.7%
Serotype 19F	490	2	6.71	4.61–9.66	0.0123	11.9%
Serotype 5	402	1	5.97	4.03–8.75	NA	NA
Serotype 6A	490	2	5.39	1.16–21.71	1.1957	91.7%
Serotype 8	490	2	5.24	2.37–11.18	0.2868	57.2%
Serotype 9N	402	2	5.15	1.68–14.68	0.5829	68%
Serotype 9V	490	2	5.15	1.68–14.68	0.5829	83.9%

**Table 6:** Meta-analysis results of the most common serogroups/serotypes in the adult CAP population

Serogroups/Serotypes	Total No. of Isolates	Number of studies (k)	Pooled Prevalence (%)	95% CI	Tau²	I²
Serotype 19A	50	1	14.00	6.82–26.57	NA	NA
Serotype 8	50	1	10.00	4.22–21.87	NA	NA
Serotype 19F	50	1	8.00	3.04–19.46	NA	NA
Serotype 3	50	1	6.00	1.95–17.02	NA	NA

### Meta-analysis of AMR/non-susceptibility patterns among the serotypes

**Fig. 6:**
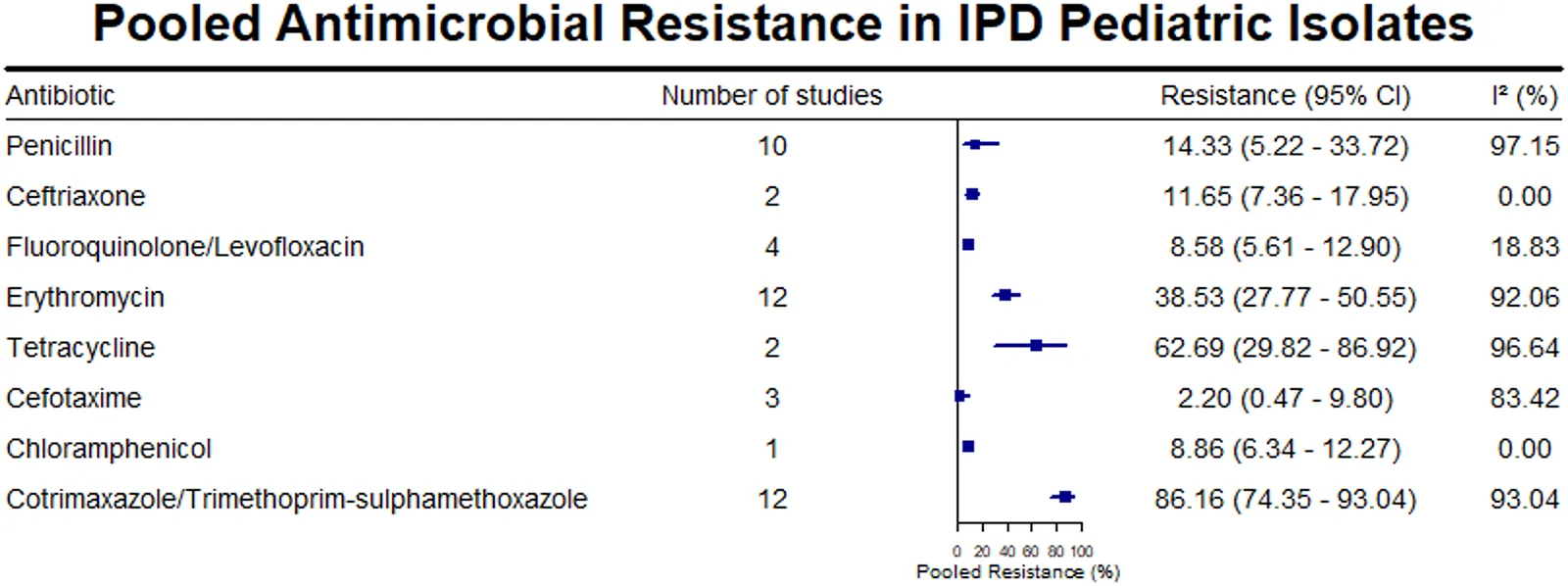
Forest plot representing the AMR/non-susceptibility pattern across the pediatric IPD population

**Fig. 7:**
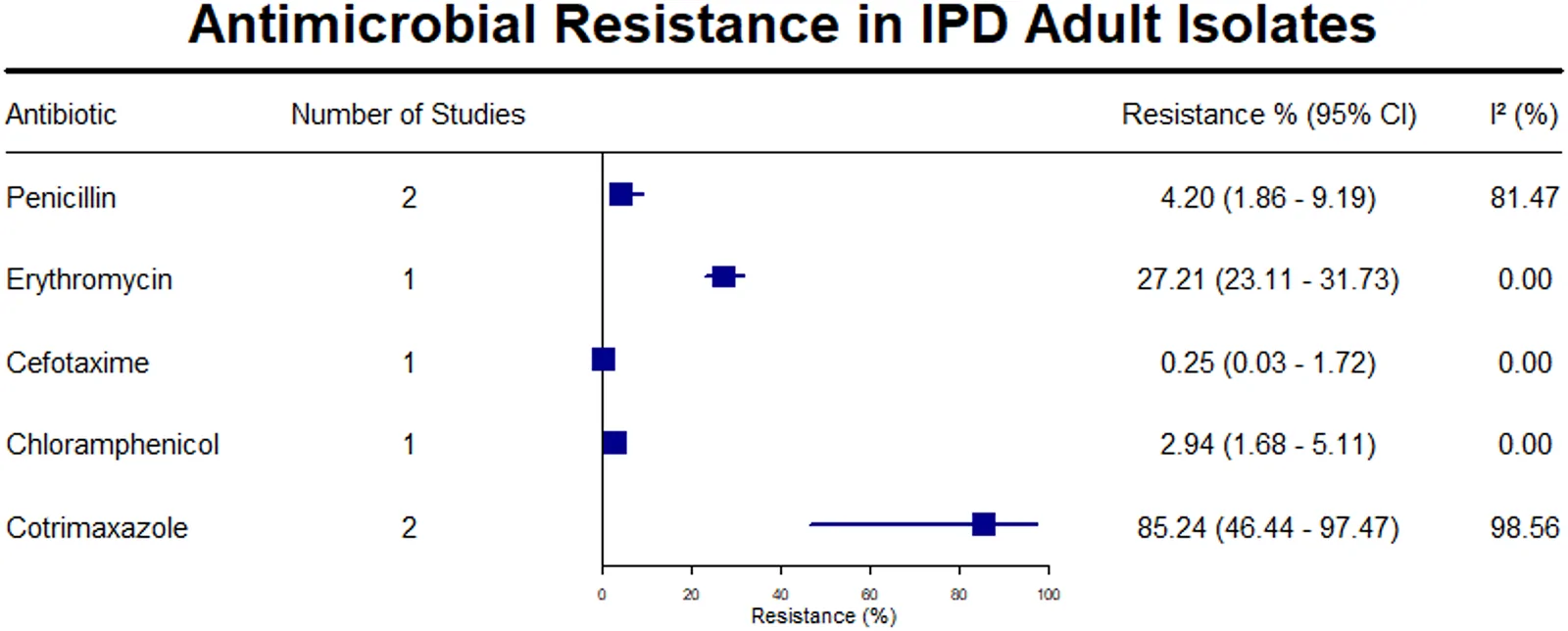
Forest plot representing the AMR/non-susceptibility pattern across the adult IPD population

**Fig. 8:**
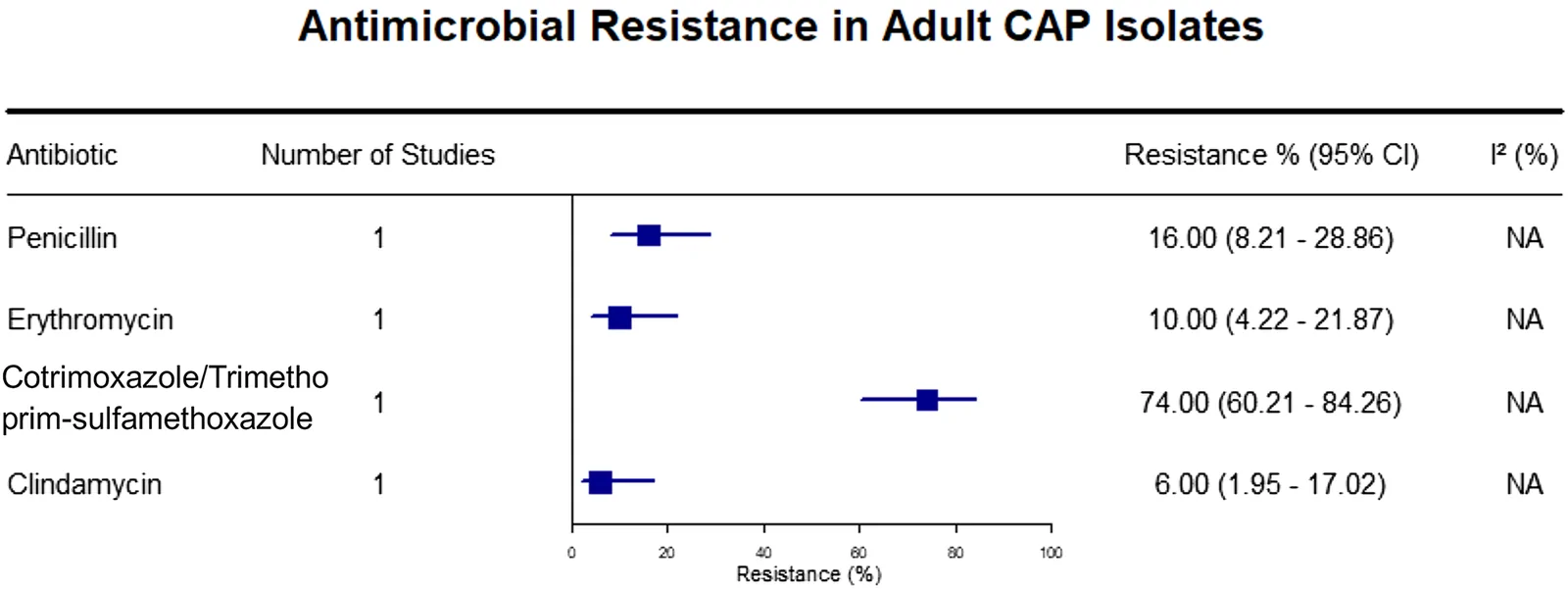
Forest plot representing the AMR/non-susceptibility pattern across the adult CAP population

AMR was analyzed across all pediatric and adult studies, revealing a concerning pattern of resistance to multiple classes of antibiotics. Among pediatric IPD studies, the highest pooled resistance rates were observed for cotrimoxazole/trimethoprim-sulfamethoxazole (86.16%, 95% CI 74.35–93.04), followed by tetracycline (62.69%, 95% CI 29.82–86.92). Erythromycin resistance was reported at 38.53% (95% CI 27.77–50.55), while resistance to β-lactams was notably lower but still significant: penicillin resistance was observed in 14.33% (95% CI 5.22–33.72), and ceftriaxone in 11.65% (95% CI 7.36–17.95). (Table 7; Fig. 6) In the adult IPD and CAP group, cotrimoxazole/ trimethoprim-sulfamethoxazole showed the highest resistance (85.24% and 74%, respectively), indicating a substantial burden of AMR among pneumococcal isolates in India. (Table 8; Figs. 7 and 8) Between-study heterogeneity was very high for several pooled resistance estimates (I² > 90% for cotrimoxazole/trimethoprim-sulfamethoxazole, penicillin, erythromycin, and tetracycline in the pediatric IPD analysis, and I² = 98.6% for cotrimoxazole/trimethoprim-sulfamethoxazole in the adult IPD analysis). At this level of heterogeneity, these pooled percentages should be interpreted with caution as overall summary measures across heterogeneous studies and not as precise prevalence estimates applicable to all settings.

**Table 7:** AMR/non-susceptibility pattern across the pediatric IPD population

Antibiotic	Number of studies (k)	*N*	Pooled Resistance/non-susceptibility (%)	95% CI (%)	I² (%)	Tau²
Penicillin	10	1187	14.33	5.22–33.72	97.15	2.9341
Ceftriaxone	2	148	11.65	7.36–17.95	0.00	0.0000
Fluoroquinolone /Levofloxacin	4	381	8.58	5.61–12.9	18.83	0.0463
Erythromycin	12	1232	38.53	27.77–50.55	92.06	0.6063
Tetracycline	2	305	62.69	29.82–86.92	96.64	0.9510
Cefotaxime	3	583	2.20	0.47–9.80	83.42	1.5324
Chloramphenicol	1	361	8.86	6.34–12.27	NA	NA
Cotrimoxazole/ Trimethoprim-sulfamethoxazole	12	1232	86.16	74.35–93.04	93.04	1.361

**Table 8:** AMR**/**non-susceptibility pattern across the adult population

Antibiotic	Number of studies (k)	*N*	Pooled Resistance/ non-susceptibility (%)	95% CI (%)	I² (%)	Tau²
**IPD**						
Penicillin	2	816	4.20	1.86–9.19	81.47	0.2991
Erythromycin	1	408	27.21	23.11–31.73	NA	NA
Cefotaxime	1	408	0.25	0.03–1.72	NA	NA
Chloramphenicol	1	408	2.94	1.68–5.11	NA	NA
Cotrimoxazole/Trimethoprim-sulfamethoxazole	2	816	85.24	46.44–97.47	98.56	1.8451
**CAP**						
Penicillin	1	50	16.00	8.21–28.86	NA	NA
Erythromycin	1	50	10.00	4.22–21.87	NA	NA
Cotrimoxazole/ Trimethoprim-sulfamethoxazole	1	50	74.00	60.21–84.26	NA	NA
Clindamycin	1	50	6.00	1.95–17.02	NA	NA

### Proportion of vaccine-type serotypes

**Fig. 9:**
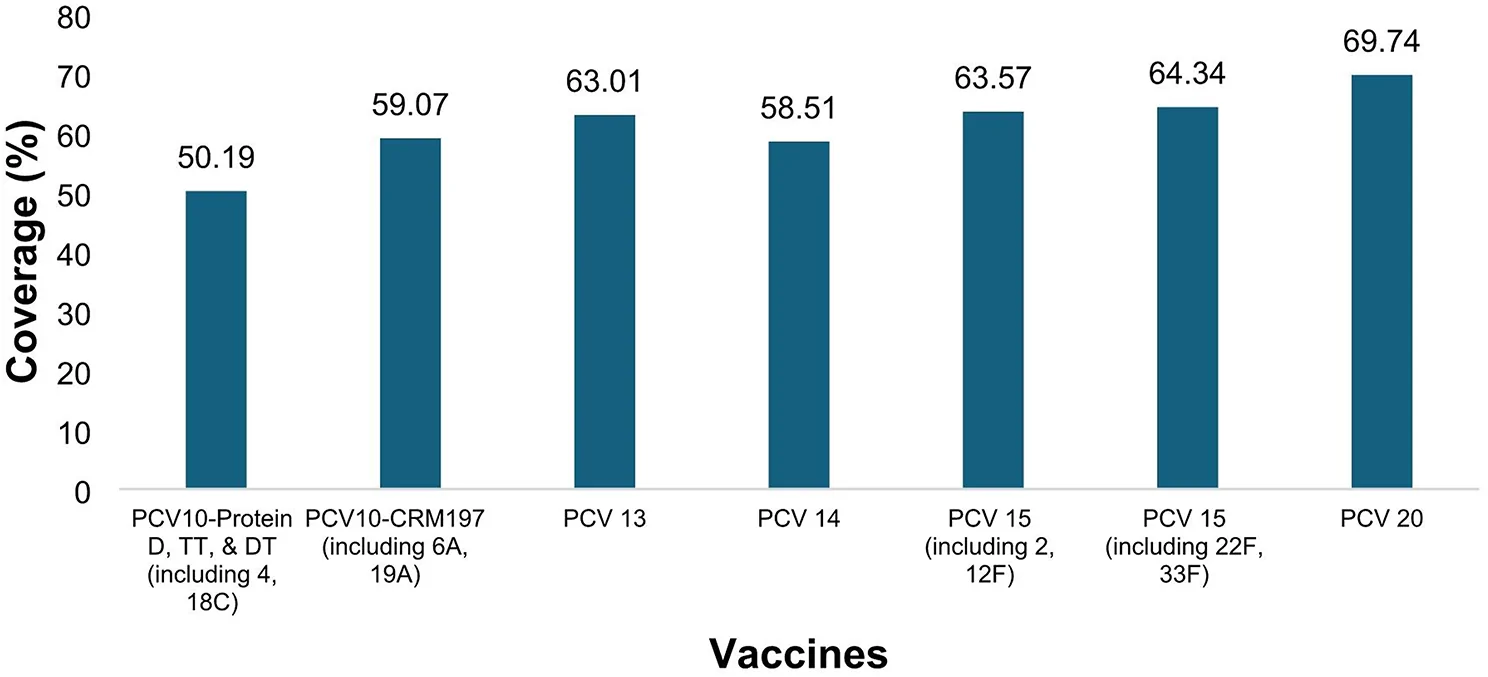
PCV serotype coverage for the overall pediatric population

Serotype coverage for currently available pneumococcal conjugate vaccines (PCVs) was also evaluated. Estimated serotype coverage varied across vaccine formulations. In the pediatric population, the estimated overall/IPD/carriage coverage was 50.19%/66%/32.21% for PCV10-Protein D, TT, & DT (including 4, 18C) and 59.07%/73.86%/40.67% for PCV10-CRM_197_ (including 6A, 19A). Corresponding estimates were 63.01%/77.67%/44.90% for PCV13, 58.51%/73.78%/40.80% for PCV14, 63.57%/78.41%/45.27% for PCV15 (including 2, 12F), 64.34%/78.83%/45.65% for (PCV15 including 22F, 33F), and 69.74%/83.04%/50.75% for PCV20. (Table 9; Fig. 9, Add. File 2-Supp. Figs. 6 and 7)

**Fig. 10:**
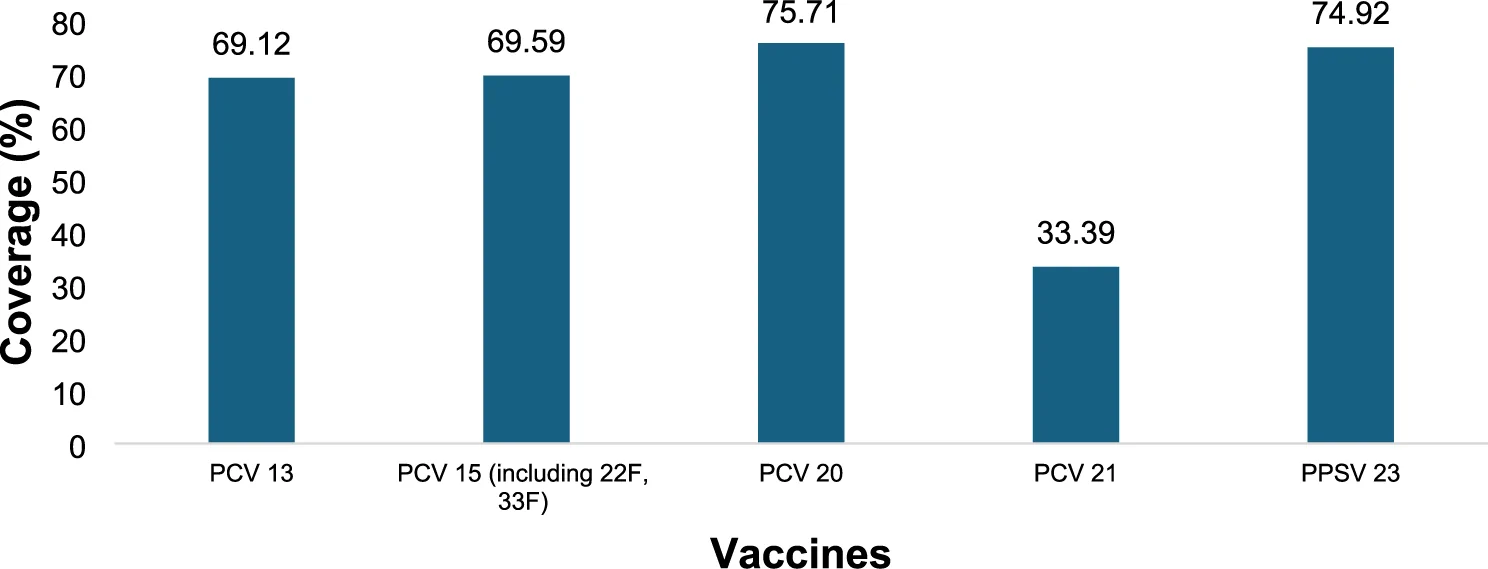
Pneumococcal vaccine* serotype coverage for the overall adult population [(*) - approved in any country for adult indication at the time of manuscript]

Among the adult population, estimated overall/IPD/CAP coverage was 69.12%/69.97%/48% for PCV13, 69.59%/70.47%/48% for PCV15 (including 22F, 33F), 75.71%/76.01%/60% for PCV20, 33.39%/31.88%/46% for PCV21, and 74.92%/74.66%/66% for PPSV23.

(Table 10; Fig. 10, Add. File 2-Supp. Figs. 8 and 9)

Despite improved coverage with higher-valent vaccines, a significant proportion of isolates were either non-vaccine serotypes (NVTs), accounting for approximately 15.67% (adults) and 13.33% (pediatric) or undifferentiated including non-typeable (NT) and non-classified comprising approximately 0.94% (adults) to 10.46% (pediatric) of all isolates, highlighting the continued risk posed by serotype replacement and underlining the importance of ongoing surveillance.

**Table 9:** PCV serotype coverage in the pediatric population(< 18 years) – Overall, IPD, and Carriage

Vaccines	Overall (%)	IPD (%)	Carriage (%)
PCV 10 (including 4/18C)	50.19	66	32.21
PCV 10 (including 6A/19A)	59.07	73.86	40.67
PCV 13	63.01	77.67	44.90
PCV 14	58.51	73.78	40.80
PCV 15 (including 2/12F)	63.57	78.41	45.27
PCV 15 (including 22F/33F)	64.34	78.83	45.65
PCV 20	69.74	83.04	50.75
Non PCV 20	19.20	3.39	8.70
**Other Serotype Category**			
Undifferentiated	10.46	3.06	25.50

**Table 10:** Pneumococcal vaccine serotype coverage in the adult population (≥18 years)– Overall, IPD, and CAP

Vaccines	Overall (%)	IPD (%)	CAP (%)
PCV 13	69.12	69.97	48
PCV 15 (including 22F/33F)	69.59	70.47	48
PCV 20	75.71	76.01	60
Non PCV 20	23.35	7.38	0.62
PCV 21^#,**^	33.39	31.88	46
PPSV 23^#^	74.92	74.66	66
**Other Serotype Category**			
Undifferentiated	0.94	1.01	0

Abbreviations: PPSV; pneumococcal polysaccharide vaccine

(^#^) – PPSV 23 is approved for > 2 years of age and PCV 21 for > 18 years of age

**) – Currently not approved or available in India as of the manuscript date

## Discussion

This meta-analysis comprehensively assessed the serotype distribution and antimicrobial resistance patterns of *S. pneumoniae* across the diverse Indian population. The findings underscore the dynamic nature of pneumococcal epidemiology in India, characterized by age-specific prevalence, emerging non-vaccine serotypes, and rising multidrug resistance.

In the pooled analysis of the pediatric population, the most prevalent pneumococcal serogroups/serotypes across the included IPD and carriage studies (≥ 5% representation) were 6, 14, 18, 19F, 6B, 19, 1, 5, 6A, 23F, 9V, 19A and 19, 23F, 19F, 19A, 6B,10, 35, 14, 6A, 10A, respectively. Notably, serogroup 10, while part of this prevalent group, is not included in the current PCV10 or PCV13 formulations. The representation of serogroup 10 in this meta-analysis was primarily driven by two carriage studies [36, 57] focusing on children aged ≤ 5 years, suggesting that this serotype may circulate subclinically in pediatric populations.

Evidence from outside India echoes these findings and underscores the potential clinical relevance of serogroup 10, particularly serotype 10A. A surveillance initiative in New Jersey, USA, using nucleic acid-based serotyping, identified serotype 10A as a dominant contributor to IPD, accounting for 25.2% of cases during a 2009 pilot. The serotype was geographically widespread but showed a localized spike in one county, with a rate ratio of 5.4 (95% CI: 2.1–11.8). A follow-up case-control study linked 10A infections to immunosuppression, asthma, and multiple comorbidities, highlighting its clinical burden in vulnerable populations [58]. 

Serotype 1, once a dominant cause of IPD, showed a declining trend, mirroring global patterns but lagging behind developed countries. The IndiaCLEN study [59] conducted from 1993 to 2008 reported a reduction of serotype 1 from 24% to 17.3% over a decade, suggesting a potential epidemiological shift. Nevertheless, it remains an important cause of disease, especially in adult IPD. Molander et al. [35] found that serotype 1 was significantly associated with pneumonia, more commonly in adults, and was isolated from patients without predisposing conditions, reinforcing its invasive nature.

A global meta-analysis of 49 studies (2007-2013) [60] identified serotype 19A as consistently prevalent across all regions, while serotypes 1 and 14 remained prominent in Europe and Latin America, and serotype 14, serotypes 6B and 19F in Africa and the Eastern Mediterranean. Among 30 carriage studies, only five found PCV serotypes comprising over 50% of isolates, highlighting a disconnect between carriage and disease. A recent regional review [61] from South and Southeast Asia (2012–2024) found serotype 14, serotypes 6A, 6B, 15B/C, 19F, and 23F common in both carriage and disease, while serotypes 1, 3, 5, 19A, 15A, 10A, and 35B were mainly associated with invasive disease. In a meta-analysis of 84 studies (1999-2022)including 16,396 isolates [62], serotypes 19F (12.6%), 23F (9.2%), and 6B (7.1%) were the most frequently identified, reaffirming the global relevance of vaccine serotypes despite emerging diversity [62]. 

Underscoring the role of serotype 14, which represents the 4th most common serotype in our overall analysis (pre-UIP), the recent (2025) [63] population-based surveillance study in Massachusetts assessed the invasive capacity (IC) of *S. pneumoniae* serotypes in children < 7 years by comparing nasopharyngeal carriage and IPD rates. Among the findings, serotype 14 was notable for its association with pneumonia, distinguishing it from most other serotypes, which primarily presented as bacteremia.

Nasopharyngeal carriage of *S. pneumoniae* remains a key reservoir for transmission and a critical precursor to invasive disease. This link was strongly affirmed by Kumar KL (2014) [52], who found that 81% of serogroups identified in carriage were also present in invasive isolates, underscoring that IPD often arises from strains within the normal nasopharyngeal flora. Studies by Rupa V [37] and Gupta et al. [47] revealed early and high rates of colonization, with nearly 50% of infants colonized by five months, driven by overcrowding, poor ventilation, and early environmental exposure. Raman et al. [54] similarly associated increased carriage of serotypes 6B and 23F with household crowding. Seasonal trends and upper respiratory infections further amplified colonization rates.

A U.S. cohort study (2013–2014) tracking 326 infants found only 11% were colonized in the first year, with a gradual increase from 0.3% at birth to 10% by 12 months. Notably, antibiotic exposure, seen in 33% of infants (67% including maternal exposure), did not influence colonization or resistance patterns [64]. In contrast, an Ethiopian case–control study of 420 children under five reported a 51.2% colonization rate, beginning as early as infancy, and peaking at over 50% in children under six months [65]. Another African study found the median time to first pneumococcal acquisition was just 63 days [66], highlighting the rapid establishment of colonization in early infancy.

Uniquely, serotype 8 presented one of the commonest serotype specifically in the 5–24 years age group. Among unique PPSV23 serotypes, serotype 8 has primarily emerged in Europe, as mentioned above. Serotype 8 has been reported to be associated with a high case fatality rate in patients with meningitis and invasive disease [67, 68]. Serotype 11 A was the 4th most common serotype in infants < 2 years and has been associated with higher mortality in patients with IPD [69, 70]. 

In the adult subgroup, serotype 3 represented the 2nd most prevalent serotype causing invasive disease. In line with our results, the WHO commissioned the Pneumococcal Serotype Replacement and Distribution Estimation (PSERENADE) project, a collaboration of more than 50 global institutions, examining the serotype distribution of residual invasive pneumococcal disease following widespread implementation of the PCV10-Protein D, TT, & DT (including 4, 18C) and PCV13 vaccines, identified serotype 3 as one of the most prevalent serotypes. It was responsible for approximately 9% of cases in children under 5 years and 14% of cases in adults aged 50 years and older, across both PCV10-Protein D, TT, & DT (including 4, 18C) and PCV13 implementation sites [71]. 

The 2025 global review of pneumococcal serotypes in adults [72] found that, despite established pediatric PCV programs in high-income countries, PCV13 serotypes, especially serotype 3, persist, with serotype 4 reemerging in the western U.S. and Canada, and serotype 8 rising in parts of Europe. In PCV10-Protein D, TT, & DT (including 4, 18C)-using countries like Finland and New Zealand, serotype 19A remained prevalent, suggesting limited cross-protection. In non–high-income countries, where PCVs are not widely used in pediatric programs, PCV serotypes still dominate, with serotype 3 and serotype 19A emerging, particularly where PCV10-Protein D, TT, & DT (including 4, 18C) is used. The introduction of PCV15, PCV20, and PCV21 is expected to alter serotype patterns, underscoring the need for continued surveillance.

The persistence of serotype 3 indicates limited herd protection likely due to its low immunogenicity [73]. Serotype 3 elicits weaker and shorter-lasting antibody responses compared to other serotypes in both PCV13 and PCV20 [72]. These reduced responses [74, 75] may contribute to inadequate mucosal immunity and limited impact on nasopharyngeal carriage [76]. Clinically, serotype 3 has been linked to higher rates of major adverse cardiovascular events (MACE) [77] and reduced quality-adjusted life years (QALY) in older adults [78], underscoring its continued public health significance.

Antimicrobial resistance remains a growing concern in India. Our pooled analysis showed the highest resistance to cotrimoxazole/trimethoprim-sulfamethoxazole, followed by macrolides and tetracyclines. Penicillin resistance was lower, though there is evidence of rising intermediate resistance. The IndiaCLEN study [59] confirmed increasing resistance in adults over time, despite initially low rates. Resistance to co-trimoxazole was noted as early as 1993, reflecting its widespread and indiscriminate use for respiratory infections. Consistent with our findings, Peela et al. [79] reported high antimicrobial resistance among *S. pneumoniae* isolates, with 66.4% resistant to trimethoprim-sulfamethoxazole, 35.1% to macrolides, and 34.3% to tetracycline.

Several studies linked specific serotypes with higher resistance rates. Kumar KL (2013) [36] observed high resistance in serogroups 6, 9, and serotype 14, while Verghese et al. found that serotypes 14, 6B, and 19F were responsible for 76.7% of penicillin-resistant meningitis cases, particularly in children under five. Nagaraj et al. [43] (2011-2013) noted that IPD among under-5-year-old children is caused by serotypes that are in large part covered by available pneumococcal vaccines and these serotypes are associated with multidrug resistance. The rise in multidrug resistance is attributed to over-the-counter antibiotic availability, subtherapeutic dosing, and prolonged use in both hospitals and the community, as discussed by Kumar KL (2014) [52].

Recent studies from India highlight rising AMR among *S. pneumoniae* in the post-vaccine era. Serotypes 19F and 19A [80], which remain prevalent, showed high multidrug resistance (up to 89.7%), with 70% of adult isolates classified as multi-drug resistant (MDR) and many exhibiting elevated penicillin MICs [81]. These findings underscore the urgent need for continued genomic surveillance and updated vaccine strategies to combat resistance and emerging serotypes.

The changing colonization dynamics post-PCV introduction have been well described in global literature. The Massachusetts communities study [82] demonstrated a rise in non-vaccine serotypes and penicillin non-susceptible strains following PCV7 introduction. Consistent with this, the Asian Network for Surveillance of Resistant Pathogens (ANSORP) [83] study reported a rise in serotype 19A in India, now a dominant non-vaccine serotype associated with penicillin non-susceptibility and multidrug resistance. The shift appears to be driven by vaccine-related serotype replacement, antibiotic pressure, and clonal expansion.

The estimated serotype coverage of currently available PCVs varied widely. Our analysis found that PCV13 showed coverage of 63.01% whereas PCV14 showed a coverage of 58.51% in pediatric population. This difference is largely attributed to the coverage of serotype 6A which is absent in PCV14. Notably, our analysis revealed serotype 6A as a commonly prevalent serotype (≥ 5% representation) in the pediatric population. Additionally, serotype 6A was the second most common serotype in the 5–24 age group, third most common in both the < 2 and sixth most common in 3–5 age groups. PCV20 provided the coverage of 69.74% in the pediatric, and increasing to 75.71% in adults.

It is important to note that some of the PCV vaccines included in our analysis are not yet approved for use in either adult [PCV15, and PCV21] or pediatric [PCV15, and PCV20] populations in India. We included these vaccines in our analysis solely for research purposes, aiming to provide insight into how future vaccine approvals might affect serotype coverage in the population.

In one study, concentration and opsonophagocytic activity (OPA) of antibodies to 6B and 6A polysaccharides were determined in serum samples from infants immunized with 3 different pneumococcal conjugate vaccines containing serotype 6B. Although pneumococcal type 6B conjugate vaccines elicit antibodies that cross-react with type 6A, not all these antibodies are functionally active against 6A [84]. 

However, significant gaps remain, particularly among non-vaccine serotypes, which accounted for 13.33% and 15.67% of isolates in pediatric and adult population and would remain uncovered despite vaccination. In studies like John et al. [41] and Samson et al. [39], up to one-third of IPD and colonizing strains were non-serotypeable or not covered by PCV13. These strains, particularly 15B, 11A, and 33B, often demonstrated multidrug resistance, suggesting the potential for future increases in disease burden.

An analysis of 8,012 IPD cases in children under 5 years of age found that PCV13 serotypes accounted for 37.4%, with higher prevalence in PCV10-Protein D, TT, & DT (including 4, 18C) countries (57.1%) compared to PCV13 countries (25.2%), mainly driven by serotype 3 and serotype 19A. PCV15 and PCV20 could expand coverage by up to 55.6% in PCV10-Protein D, TT, & DT (including 4, 18C) countries and 38.2% in PCV13 countries, targeting additional serotypes like 10A, 12F, 22F, and 33F [85]. 

Similarly, in adults aged ≥ 65 across 33 high-income countries, PCV13 serotypes caused 33.7% of IPD, with serotype 3 (14.9%) and serotype 19A (7.0%) most common. PCV15 and PCV20 would offer broader protection, extending coverage by 10.4% and 32.9%, respectively, particularly against serotype 8, serotypes 22F, 12F, and 11A. Notably, PPSV23 use did not reduce disease from its covered serotypes [86]. 

Finally, while existing adult vaccine recommendations in India include either a single dose of PCV20 or a sequencing with PCV13 and PPSV23 for those over 50 years of age or with risk factors, their uptake remains limited due to poor awareness and insufficient local data. However, the incidence or proportion of pneumococcal diseases caused by serotypes included in PPSV23 has not declined, possibly because of the limitations of the direct effectiveness of PPSV23 or low vaccination coverage among target populations. Limitations in diagnostic infrastructure may underestimate the true disease burden and serotype diversity. Most serotyping methods rely on culture-positive isolates [56], whereas many clinically suspected IPD cases are culture-negative due to prior antibiotic exposure or delayed presentation. PCR-based detection can identify invasive serotypes missed by culture alone, indicating that surveillance using molecular diagnostics is essential for accurate burden estimation [55]; however, cost and availability limit it’s widespread application. 

The strength of our meta-analysis lies in its comprehensive evaluation to date of *S. pneumoniae* serotype distribution and antimicrobial resistance across India’s diverse regions and age groups. To our knowledge, this is the first meta-analysis from India to systematically examine pneumococcal serotypes and AMR patterns in adults. Equally significant is our age-stratified pediatric subgroup analysis which illustrates the shifting landscape of pneumococcal disease burden across childhood stages. By incorporating both invasive disease and nasopharyngeal carriage data, the analysis captures the full spectrum of pneumococcal epidemiology and provides critical, population-specific insights for vaccine policy formulation and implementation.

Certain limitations merit consideration. Sensitivity analyses and publication bias assessments were not performed, as most analyses included too few studies to yield reliable or interpretable results. AMR comparability was limited by variation in susceptibility testing practices and CLSI breakpoint versions over the nearly two-decade study period. As MIC-level data were not consistently available, isolates could not be reclassified using a single contemporary breakpoint; therefore, pooled estimates reflect resistance/non-susceptibility as reported in the original studies. The analysis has not accounted for the vaccination context in each Indian state where surveillance was conducted, including whether vaccination was delivered through a public or private program, the age groups covered, vaccine uptake where available, and the relevant implementation period because these details are not available in the published studies. Also, the correlation of any specific serotype with antibiotic resistance has not been included in the analysis as the details have not been provided in the published studies. Hence, it is difficult to determine whether the reported resistance patterns reflect vaccine implementation impact or other differences among studies. Study heterogeneity, due to differences in design, geography, and diagnostic methods, may affect pooled estimates. Most data are predominantly from southern India, limiting national representativeness. Reliance on culture-based diagnostics likely under-detects non-typeable or PCR-only serotypes, particularly post-antibiotic exposure. In the pediatric dataset, only 10 of 23 studies include serotype data after 2015, compared to 3 of 5 in the adult dataset. This indicates a greater proportion of older data in the pediatric group, highlighting the need for more recent serotype studies in children. The percentage of undifferentiated isolates would have been incorporated into the respective PCV serotype coverage estimates if the isolates had been clearly identifiable. PCV13 was the first vaccine to be introduced in the UIP in a few states of India. This was followed by the introduction of PCV10-CRM_197_ (including 6A, 19A) in 2021, which was the first indigenous PCV. In 2024, PCV 14 was licensed for use under the UIP rollout. Since these vaccines have different serotype coverage, it will tend to have an impact on the serotype prevalence seen in India [87]. Most of the studies included (24 / 26) are from the pre-UIP period. In addition, several estimates - including the entire adult CAP subgroup and the leading pediatric IPD serogroups (6, 18, and 19) - were derived from single studies, for which between-study heterogeneity could not be assessed and the confidence intervals reflect within-study sampling variability only; these are hypothesis-generating rather than robust pooled prevalences. Pooled AMR estimates also showed very high between-study heterogeneity (I² > 90% for most antibiotics), so these values should be interpreted as approximate indicators of resistance burden rather than precise national estimates.The widespread reporting of non-specific serotype groups across studies may limit accurate assessment of PCV program effectiveness and the estimated coverage of newer higher-valency PCVs. Therefore, countries should prioritize serotype-specific identification methods and report individual serotype data rather than aggregated distributions aligned with specific PCV formulations [88].

## Conclusion

This meta-analysis highlights the evolving landscape of pneumococcal serotypes in India, influenced by vaccine introduction, microbial ecology, and antimicrobial resistance trends. With the introduction of PCV in the India UIP, there could be persistence of certain non-vaccine serotypes, characterized by an emergence of multidrug-resistant strains. There is a need for continuous surveillance of serotypes and AMR patterns in pneumococcal disease to guide the national vaccination policies and choice of PCVs. The vaccination strategy should be dynamic, based on changing serotype patterns as well as other factors such as real-world evidence, AMR data, and vaccine-related factors.

The burden of pneumococcal disease remains significant across all age groups in India, and higher-valency vaccines hold promise for closing existing protection gaps. However, effective disease control requires more than just expanded valency; it demands broader vaccine access, especially among adults, older populations, and high-risk groups, where uptake remains low. Awareness campaigns and healthcare infrastructure must prioritize these populations to realize the full potential of pneumococcal vaccination.

## Supplementary Information

Below is the link to the electronic supplementary material.

Supplementary Material 1

Supplementary Material 2

Supplementary Material 3

## Data Availability

All data generated or analysed during this study are included in this published article [and its supplementary information files].

## Abbreviations

AMR: Antimicrobial Resistance
ANSORP: Asian Network for Surveillance of Resistant Pathogens
Auro/Tr: Aurobindo/Tergene Biotech
CAP: Community-Acquired Pneumonia
CAPITA: Community-Acquired Pneumonia Immunization Trial in Adults
CLSI: Clinical and Laboratory Standards Institute
CI: Confidence Interval
CPS: Capsular Polysaccharide Synthesis
CSF: Cerebrospinal Fluid
DL: DerSimonian and Laird MethodIC: Invasive Capacity
IPD: Invasive Pneumococcal Disease
LCI: Lower Confidence Interval
MeSH: Medical Subject Headings
MICs: Minimum Inhibitory Concentrations
MDR: Multidrug Resistance
NOS: Newcastle-Ottawa Scale
NVT: Non-Vaccine Serotypes
NT: Non-Typeable
OPA: Opsonophagocytic Activity
PCR: Polymerase Chain Reaction
PCV: Pneumococcal Conjugate Vaccine
PLOGIT: Logit transformation
PSERENADE: Pneumococcal Serotype Replacement and Distribution Estimation
QALY: Quality-Adjusted Life Years
REML: Restricted Maximum-Likelihood
UCI: Upper Confidence Interval
UIP: Universal Immunization Program
