# Supplementary material for: Mapping the pneumococcal disease landscape in India: a meta-analysis of literature from 2007 to 2024 on serotype prevalence and antimicrobial resistance patterns
Source: BMC Infect Dis. 2026 Nov 3;26:1989. doi: 10.1186/s12879-026-14389-x (PMC13761146; doi:10.1186/s12879-026-14389-x)
Supplement: Supplementary file 1 [file 12879_2026_14389_MOESM1_ESM.docx]

**Table 1: Key characteristics of the included studies**

| S. No. | First Author | Publication year | Period of study | Region of India | Age | Cohort | Type of study | Method of serotyping | Method of AMR assessment | Most common serotype |
| --- | --- | --- | --- | --- | --- | --- | --- | --- | --- | --- |
| 1. | Wattal C(1) | 2007 | 4 months* | North | 3 months to 3 years | Carriage (Healthy) | Single centre, prospective, hospital based | Quellung reaction | Disk diffusion and E test methods | 19 |
| 2. | Nandan TM(2) | 2013 | 2008-2009 | South | ≥3 months ≤5 years | Carriage (Healthy) | Single centre, prospective, hospital based | Quellung reaction | Kriby-Bauer disc diffusion method | 19 |
| 3. | Thomas K(3) | 2013 | 1993-2008 | North, South, West | >15 years | IPD | INCLEN study, multisite, prospective, hospital surveillance | Quellung technique or coagglutination technique | Kirby-Bauer disc diffusion method | 1 |
| 4. | Kumar KL(4) | 2013 | 2009-2011 | South | 28 days to <5 years | IPD | Single centre, prospective, hospital based | Quellung reaction | Antibiogram performed by microdilution procedure | 6 |
| 5. | Kumar KL(5) | 2014 | 2010-2011 | South | 3 months to <5 years | Carriage (Healthy) | Single centre, prospective, hospital based | Quellung reaction | E test | 19 |
| 6. | Rupa V(6) | 2014 | 2009-2011 | South |  | Carriage (Healthy and Upper Respiratory Infection^#^) | Single centre, prospective, hospital based | Quellung reaction | Kirby Bauer disk diffusion method | 19 |
| 7. | Balaji V(7) | 2015 | 2007-2013 | South | 60 days to 5 years | IPD | Single centre, prospective, hospital based | Co-agglutination technique, reconfirmed with multiplex PCR. | Agar dilution method | 14 |
| 8. | Nisarga R(8) | 2015 | 2009-2011 | South | 28 days to <60 months | IPD | Multi hospital, prospective, surveillance study | Quellung reaction | Microbroth dilution and/or E-test | 6A |
| 9. | Gopi T(9) | 2016 | 2010-2011 | South | <5 years | IPD | Single centre, laboratory-based study | Co-agglutination technique | Broth microdilution | 19F |
| 10. | Vandana G(10) | 2016 | __ | South, North, East, West | 6 weeks to 5 years | IPD | PIDOPS study, multisite, prospective, surveillance study | Quellung and PCRSeqTyping | Kirby-Bau er’ s disc diffusion method | 1 |
| 11. | Manoharan A(11) | 2016 | 2011-2015 | South, North, East, West | <5 years | IPD | Prospective hospital-based and retrospective laboratory-based surveillance study | Quellung reaction | E test | 14,1 |
| 12. | Nagaraj S(12) | 2017 | 2011-2013 | South | <5 years | IPD | Part of ASIP study, single-centre, prospective, hospital based | Quellung reaction | Kirby-Bauer disc diffusion method and E test | 6B, 19F |
| 13. | Raman R(13) | 2017 | 2012-2013 | South | 6 months to 5 years | Carriage (Healthy) | Single centre, prospective, hospital-based | Latex agglutination | Kirby-Bauer method and E test | 6B |
| 14. | Wattal C(14) | 2017 | 2013-2015 | North | ≥50 years | CAP | Single centre, prospective, hospital-based | Quellung reaction | Kirby-Bauer method and E test | 19A |
| 15. | John J(15) | 2018 | 2007-2016 | South | <5 years | IPD | Single centre, retrospective, hospital-based | Co-agglutination reaction and sequential multiplex PCR | Agar dilution method for MIC | 11A, 15B |
| 16. | Jayaraman R(16) | 2018 | 2007-2017 | South | ≥18 years | IPD | Single centre, prospective, laboratory based | Co agglutination technique | Agar dilution method and E test | 1 |
| 17. | Sutcliffe CG(17) | 2019 | 2016-2017 | North | 2-59 months | Carriage (Clinical Pneumonia) | Part of BASIS study, single centre, cross-sectional study | Conventional culture and sequential multiplex PCR | E test | 6A |
| 18. | Samson D(18) | 2020 | __ | South | 8years old | Carriage (Otitis Media and Upper Respiratory Infection^#^) | Single centre, prospective cohort study at 8 years follow-up | Quellung method and customized sequential multiplex PCR | __ | 6A |
| 19. | Kumar S(19) | 2021 | 2012-2014 | North | <10 years | Carriage (Upper Respiratory Tract^#^) | Rural community-based surveillance study | Quellung reaction and PCR | Kirby-Bauer disc diffusion method | 6B/C |
| 20. | Girish Kumar CP(20) | 2021 | 2016-2018 | South | 1-59 months | IPD | Multisite, prospective, laboratory based, surveillance study | Quellung reaction | E-test | 14 |
| 21. | Verghese R(21) | 2021 | 2012-2018 | South | <5 years | IPD | Single centre, retrospective, laboratory-based study | Quellung reaction, modified version of multiplex PCR | E-test | 19F |
| 22. | Varghese R(22) | 2021 | 2014-2019 | South, North | All ages | IPD | Multisite, prospective, laboratory based, surveillance study | Co-agglutination method | E-test | 14, 19F |
| 23. | Jayaraman Y(23) | 2021 | 2012-2016 | South, North, East | <5 years | IPD | Multi-centric, cross-sectional, hospital-based, sentinel surveillance study | Latex agglutination, and real-time PCR | E test | 14 |
| 24. | Gupta P(24) | 2023 | July to August 2019 | North | 2-59 months | Carriage (Healthy) | Multi-centric, cross-sectional, laboratory-based | Quellung reaction | E test | 19F |
| 25. | Verma N(25) | 2023 | 2017-2022 | North | 2-59 months | Carriage (Healthy) and CAP | Multi-site, prospective, case control study | Quellung reaction | __ | 6A |
| 26. | Rafiqullah IM(26) | 2024 | 1991-2020 | South | <5 years | IPD | Retrospective archived isolate collection, laboratory-based study | Quellung reaction | E-test | 6B, 19A |

** exact year of study not specified*

*^#^ No confirmed disease type*

***Abbreviations:*** *CLSI; Clinical and Laboratory Standards Institute, INCLEN; International Clinical Epidemiology Network, ANSORP; Asian Network for Surveillance of Resistant Pathogens, MIC; minimal inhibitory concentration, PIDOPS; Pan India distribution of pneumococcal serotypes, ASIP; Alliance for Surveillance of Invasive Pneumococci, PCR; polymerase chain reaction, BASIS; Baseline Assessment for S. pneumoniae of India Serotypes.*

*Number of studies including IPD population- 15/26 (57.69%); Number of studies including carriages- 9/26 (34.61%); Number of studies including CAP- 1/26 (3.85%); Unclear distinction of study group- 1/26(3.85%).*

*3 studies primarily included adults, 22 included pediatric, and the remaining 1 focused on both adult & pediatric with split data.*

***References:***

1.	Wattal C, Oberoi JK, Pruthi PK, Gupta S. Nasopharyngeal carriage of Streptococcus pneumoniae. Indian J Pediatr. 2007 Oct;74(10):905–7.

2.	PNEUMOCOCCAL CARRIAGE RATE IN CHILDREN AND THEIR ANTIMICROBIAL SUSCEPTIBILITY PATTERNS IN AN URBAN SET UP [Internet]. [cited 2025 May 17]. Available from: https://www.europub.co.uk/articles/nbsppneumococcal-carriage-rate-in-children-and-their-antimicrobial-susceptibility-patterns-in-an-urban-set-up-A-111820

3.	Thomas K, Mukkai Kesavan L, Veeraraghavan B, Jasmine S, Jude J, Shubankar M, et al. Invasive pneumococcal disease associated with high case fatality in India. Journal of Clinical Epidemiology. 2013 Jan;66(1):36–43.

4.	Kumar K.L. R, Ganaie F, Ashok V. Circulating Serotypes and Trends in Antibiotic Resistance of Invasive Streptococcus Pneumoniae from Children under Five in Bangalore. J Clin Diagn Res. 2013 Dec;7(12):2716–20.

5.	Kumar KLR, Ashok V, Ganaie F, Ramesh AC. Nasopharyngeal carriage, antibiogram & serotype distribution of Streptococcus pneumoniae among healthy under five children. INDIAN J MED RES. 2014;

6.	Rupa V, Isaac R, Jalagandeeswaran R, Manoharan A, Rebekah G. Epidemiology of nasopharyngeal colonization by S. pneumoniae in Indian infants in the first 2 years of life. International Journal of Pediatric Otorhinolaryngology. 2014 Oct;78(10):1701–6.

7.	Balaji V, Jayaraman R, Verghese V, Baliga P, Kurien T. Pneumococcal serotypes associated with invasive disease in under five children in India & implications for vaccine policy. Indian J Med Res. 2015;142(3):286.

8.	Nisarga R, Premalatha R, Shivananda, Ravikumar KL, Shivappa U, Gopi A, et al. Hospital-based surveillance of invasive pneumococcal disease and pneumonia in South Bangalore, India. Indian Pediatr. 2015 Mar;52(3):205–11.

9.	Gopi T, Ranjith J, Anandan S, Balaji V. Epidemiological characterisation of *Streptococcus pneumoniae* from India using multilocus sequence typing. Indian Journal of Medical Microbiology. 2016 Jan 1;34(1):17–21.

10.	Vandana G, Feroze AG, Geetha N, Avid H, Ravi Kumar KL. Pan India distribution of pneumococcal serotypes (PIDOPS) causing invasive pneumococcal disease and pneumonia in children between 6 weeks and 5 years and their antimicrobial resistance – Phase I. Pediatric Infectious Disease. 2016 Apr 1;8(2):47–51.

11.	Manoharan A, Manchanda V, Balasubramanian S, Lalwani S, Modak M, Bai S, et al. Invasive pneumococcal disease in children aged younger than 5 years in India: a surveillance study. The Lancet Infectious Diseases. 2017 Mar;17(3):305–12.

12.	Nagaraj S, Kalal BS, Manoharan A, Shet A. Streptococcus pneumoniae serotype prevalence and antibiotic resistance among young children with invasive pneumococcal disease: experience from a tertiary care center in South India. GERMS. 2017 June 1;7(2):78–85.

13.	Raman R, Sankar J, Putlibai S, Raghavan V. Demographic Profile of Healthy Children with Nasopharyngeal Colonisation of Streptococcus pneumoniae: A Research Paper. Indian Journal of Medical Microbiology. 2017 Oct;35(4):607–9.

14.	Wattal C, Goel N, Byotra SP. Prevalence of Pneumococcal Serotypes in Adults ≥50 Years of Age. Indian Journal of Medical Microbiology. 2017 Jan 1;35(1):95–100.

15.	John J, Varghese R, Lionell J, Neeravi A, Veeraraghavan B. Non-vaccine Pneumococcal Serotypes Among Children with Invasive Pneumococcal Disease. Indian Pediatr. 2018 Oct;55(10):874–6.

16.	Jayaraman R, Varghese R, Kumar JL, Neeravi A, Shanmugasundaram D, Ralph R, et al. Invasive pneumococcal disease in Indian adults: 11 years’ experience. Journal of Microbiology, Immunology and Infection. 2019 Oct;52(5):736–42.

17.	Sutcliffe CG, Shet A, Varghese R, Veeraraghavan B, Manoharan A, Wahl B, et al. Nasopharyngeal carriage of Streptococcus pneumoniae serotypes among children in India prior to the introduction of pneumococcal conjugate vaccines: a cross-sectional study. BMC Infect Dis. 2019 Dec;19(1):605.

18.	Samson D, Rupa V, Veeraraghavan B, Varghese R, Isaac R, L J. Follow up of a birth cohort to identify prevalence and risk factors for otitis media among Indian children in the eighth year of life. International Journal of Pediatric Otorhinolaryngology. 2020 Oct;137:110201.

19.	Kumar S, Purakayastha DR, Kapil A, Saha S, Dawood FS, Das BK, et al. Carriage rates and antimicrobial sensitivity of pneumococci in the upper respiratory tract of children less than ten years old, in a north Indian rural community. Ricci S, editor. PLoS ONE. 2021 Feb 4;16(2):e0246522.

20.	Girish Kumar CP, Sukumar B, Veeraraghavan B, Gajendran S, Daniel JL, Varghese R, et al. Surveillance of bacterial pneumonia and other invasive bacterial diseases among under-five children in India, 2016–2018. International Journal of Infectious Diseases. 2020 Dec;101:439–40.

21.	Varghese R, Neeravi A, Jacob JJ, Vasudevan K, Kumar JL, Subramanian N, et al. Comparative genomics of invasive Streptococcus pneumoniae CC320/271 serotype 19F/19A before the introduction of pneumococcal vaccine in India. Mol Biol Rep. 2021 Apr;48(4):3265–76.

22.	Varghese R, Daniel JL, Neeravi A, Baskar P, Manoharan A, Sundaram B, et al. Multicentric Analysis of Erythromycin Resistance Determinants in Invasive Streptococcus pneumoniae; Associated Serotypes and Sequence Types in India. Curr Microbiol. 2021 Aug;78(8):3239–45.

23.	Jayaraman Y, Veeraraghavan B, Girish Kumar CP, Sukumar B, Rajkumar P, Kangusamy B, et al. Hospital-based sentinel surveillance for bacterial meningitis in under-five children prior to the introduction of the PCV13 in India. Vaccine. 2021 June;39(28):3737–44.

24.	Gupta P, Awasthi S, Gupta U, Verma N, Rastogi T, Pandey Ak, et al. Nasopharyngeal Carriage of Streptococcus pneumoniae Serotypes Among Healthy Children in Northern India. Curr Microbiol. 2023 Jan;80(1):41.

25.	Verma N, Gupta P, Pandey AK, Awasthi S. Nasopharyngeal carriage of Streptococcus pneumoniae serotypes among sick and healthy children in northern India: A case-control study. Vaccine. 2023 Oct 20;41(44):6619–24.

26.	Rafiqullah IM, Varghese R, Hellmann KT, Velmurugan A, Neeravi A, Kumar Daniel JL, et al. Pneumococcal population genomics changes during the early time period of conjugate vaccine uptake in southern India. Microbial Genomics [Internet]. 2024 Feb 5 [cited 2025 Feb 6];10(2). Available from: https://www.microbiologyresearch.org/content/journal/mgen/10.1099/mgen.0.001191
