# Supplementary material for: Mapping the pneumococcal disease landscape in India: a meta-analysis of literature from 2007 to 2024 on serotype prevalence and antimicrobial resistance patterns
Source: BMC Infect Dis. 2026 Nov 3;26:1989. doi: 10.1186/s12879-026-14389-x (PMC13761146; doi:10.1186/s12879-026-14389-x)
Supplement: Supplementary file 3 [file 12879_2026_14389_MOESM3_ESM.docx]

**Figure 1: Risk of Bias (RoB) assessment**

**Table 1: Serogroups/Serotypes distribution across Pediatric population – IPD**

| **Serogroups/ Serotypes** | **Total No. of Isolates** | **Number of studies** | **Pooled Prevalence (%)** | **95% CI** | **Tau²** | **I²** |
| --- | --- | --- | --- | --- | --- | --- |
| **Serotype 1** | 1163 | 10 | **7.09** | 4.71-10.55 | 0.2683 | 64.7 |
| Serotype 2 | 375 | 2 | 2.48 | 0.52-11.07 | 0.8061 | 55.7 |
| Serotype 3 | 545 | 5 | 1.74 | 0.99-3.04 | 0 | 0 |
| Serotype 4 | 820 | 6 | 2.38 | 1.14-4.90 | 0.3654 | 44.3 |
| **Serotype 5** | 1189 | 10 | **6.66** | 4.52-9.70 | 0.2243 | 57.8 |
| **Serogroup 6** | 40 | 1 | **25.00** | 14.01-40.54 | NA | NA |
| **Serotype 6A** | 1080 | 9 | **6.27** | 4.50-8.67 | 0.1011 | 37.8 |
| **Serotype 6B** | 1301 | 11 | **10.17** | 8.50-12.11 | 0.01 | 9 |
| Serotype 6C | 685 | 4 | 0.96 | 0.40-2.30 | 0 | 0 |
| Serotype 7A | 170 | 1 | 0.59 | 0.08-4.05 | NA | NA |
| Serotype 7B | 361 | 1 | 0.28 | 0.04-1.94 | NA | NA |
| Serotype 7C | 361 | 1 | 1.39 | 0.58-3.28 | NA | NA |
| Serotype 7F | 690 | 4 | 1.76 | 0.38-7.78 | 1.9494 | 82.5 |
| Serotype 8 | 915 | 5 | 0.61 | 0.25-1.45 | 0 | 0 |
| **Serogroup 9** | 40 | 1 | **5.00** | 1.25-17.91 | NA | NA |
| Serotype 9A | 361 | 1 | 0.83 | 0.27-2.54 | NA | NA |
| Serotype 9N | 284 | 2 | 0.72 | 0.18-2.83 | 0 | 0 |
| **Serotype 9V** | 1163 | 10 | **5.51** | 4.07-7.41 | 0.0516 | 20.9 |
| Serogroup 10 | 40 | 1 | 2.50 | 0.35-15.73 | NA | NA |
| Serotype 10A | 845 | 6 | 2.21 | 1.07-4.48 | 0.3358 | 41.7 |
| Serotype 10C | 206 | 2 | 1.27 | 0.28-5.70 | 0.2224 | 17.9 |
| Serotype 10F | 753 | 4 | 1.44 | 0.77-2.65 | 0 | 0 |
| Serotype 11A | 793 | 5 | 2.40 | 1.10-5.17 | 0.3968 | 50.1 |
| Serotype 11D | 108 | 1 | 0.93 | 0.13-6.28 | NA | NA |
| Serotype 12F | 531 | 2 | 0.68 | 0.17-2.66 | 0.3046 | 28.8 |
| Serotype 13 | 332 | 2 | 0.96 | 0.31-2.95 | 0 | 0 |
| **Serotype 14** | 1341 | 12 | **14.15** | 11.97-16.64 | 0.0252 | 23.5 |
| Serogroup 15 | 202 | 2 | 2.00 | 0.75-5.20 | 0 | 0 |
| Serotype 15A | 545 | 3 | 1.60 | 0.62-4.09 | 0.2375 | 31.6 |
| Serotype 15B | 793 | 5 | 2.38 | 1.15-4.87 | 0.3434 | 48.9 |
| Serotype 15C | 685 | 4 | 1.73 | 0.21-12.66 | 3.8946 | 87 |
| Serotype 15F | 361 | 1 | 0.28 | 0.04-1.94 | NA | NA |
| Serotype 16F | 531 | 2 | 0.57 | 0.18-1.74 | 0 | 0 |
| Serogroup 17 | 108 | 1 | 0.93 | 0.13-6.28 | NA | NA |
| Serotype 17F | 847 | 5 | 0.74 | 0.31-1.76 | 0 | 0 |
| **Serogroup 18** | 40 | 1 | **12.50** | 5.30-26.72 | NA | NA |
| Serotype 18A | 673 | 4 | 0.80 | 0.30-2.11 | 0 | 0 |
| Serotype 18B | 361 | 1 | 0.28 | 0.04-1.94 | NA | NA |
| Serotype 18C | 726 | 5 | 3.31 | 1.46-7.35 | 0.6074 | 67.7 |
| Serotype 18F | 505 | 3 | 1.25 | 0.49-3.19 | 0.0941 | 12.9 |
| **Serogroup 19** | 40 | 1 | **10.00** | 3.80-23.79 | NA | NA |
| **Serotype 19A** | 1263 | 9 | **5.28** | 3.62-7.64 | 0.1574 | 50 |
| Serotype 19B | 659 | 4 | 1.19 | 0.44-3.17 | 0.2963 | 27.8 |
| **Serotype 19F** | 1147 | 11 | **11.39** | 9.31-13.87 | 0.0401 | 29.9 |
| Serogroup 20 | 278 | 2 | 1.81 | 0.31-9.95 | 1.1191 | 63.9 |
| Serotype 21 | 375 | 2 | 2.10 | 0.25-15.54 | 1.751 | 71.3 |
| Serotype 22A | 324 | 3 | 1.46 | 0.55-3.82 | 0 | 0 |
| Serotype 22F | 469 | 2 | 1.14 | 0.48-2.72 | 0 | 0 |
| Serotype 23A | 645 | 3 | 0.80 | 0.26-2.38 | 0.1858 | 18.7 |
| **Serotype 23F** | 1119 | 7 | **5.89** | 4.63-7.47 | 0 | 0 |
| Serotype 24A | 361 | 1 | 0.28 | 0.04-1.94 | NA | NA |
| Serotype 24B | 284 | 2 | 1.07 | 0.34-3.25 | 0 | 0 |
| Serotype 24F | 685 | 4 | 0.95 | 0.43-2.10 | 0 | 0 |
| Serotype 25A | 361 | 1 | 0.28 | 0.04-1.94 | NA | NA |
| Serotype 27 | 685 | 4 | 1.28 | 0.37-4.29 | 0.8834 | 55.5 |
| Serotype 28F | 361 | 1 | 0.28 | 0.04-1.94 | NA | NA |
| Serotype 29 | 847 | 5 | 0.90 | 0.41-1.99 | 0 | 0 |
| Serotype 31 | 361 | 1 | 0.28 | 0.04-1.94 | NA | NA |
| Serogroup 33 | 531 | 2 | 0.57 | 0.18-1.74 | 0 | 0 |
| Serotype 33A | 361 | 1 | 0.28 | 0.04-1.94 | NA | NA |
| Serotype 33B | 361 | 1 | 0.28 | 0.04-1.94 | NA | NA |
| Serotype 33C | 324 | 3 | 1.60 | 0.67-3.80 | 0 | 0 |
| Serotype 33F | 554 | 4 | 1.67 | 0.87-3.17 | 0 | 0 |
| Serotype 34 | 531 | 2 | 0.57 | 0.18-1.74 | 0 | 0 |
| Serotype 35A | 361 | 1 | 0.55 | 0.14-2.19 | NA | NA |
| Serotype 35B | 361 | 1 | 0.55 | 0.14-2.19 | NA | NA |
| Serotype 35F | 375 | 2 | 2.60 | 0.68-9.47 | 0.525 | 45.7 |
| Serotype 36 | 361 | 1 | 0.28 | 0.04-1.94 | NA | NA |
| Serotype 38 | 693 | 3 | 0.73 | 0.30-1.74 | 0 | 0 |
| Serotype 39 | 284 | 2 | 0.72 | 0.18-2.83 | 0 | 0 |
| Serotype 40 | 361 | 1 | 0.28 | 0.04-1.94 | NA | NA |
| Serotype 41F | 361 | 1 | 0.28 | 0.04-1.94 | NA | NA |
| Serotype 45 | 523 | 2 | 0.41 | 0.10-1.64 | 0 | 0 |
| Serotype 47F | 284 | 2 | 1.22 | 0.39-3.71 | 0 | 0 |
| Serotype 48 | 361 | 1 | 0.55 | 0.14-2.19 | NA | NA |
| NVT | 107 | 1 | 26.17 | 18.72-35.29 | NA | NA |
| Non Typeable | 468 | 2 | 1.48 | 0.71-3.07 | 0 | 0 |

**Table 2: Serogroups/Serotypes distribution across Pediatric population – Carriage**

| **Serogroups/ Serotypes** | **Total No. of Isolates** | **Number of studies** | **Pooled Prevalence (%)** | **95% CI** | **Tau²** | **I²** |
| --- | --- | --- | --- | --- | --- | --- |
| Serotype 1 | 1163 | 4 | 3.75 | 1.34-10.01 | 0.6176 | 55 |
| Serotype 2 | 375 | 1 | 0.64 | 0.21-1.95 | NA | NA |
| Serotype 3 | 545 | 6 | 1.65 | 0.41-6.34 | 2.3184 | 80.3 |
| Serotype 4 | 820 | 5 | 2.45 | 0.96-6.12 | 0.7994 | 70.2 |
| Serotype 5 | 1189 | 2 | 0.8 | 0.30-2.10 | 0 | 0 |
| Serogroup 6 | 40 | 7 | 3.66 | 1.58-8.24 | 0.7937 | 66.4 |
| **Serotype 6A** | 701 | 4 | **5.64** | 3.19-9.79 | 0.2206 | 61.5 |
| **Serotype 6B** | 526 | 4 | **6.56** | 1.51-24.40 | 2.0597 | 92.4 |
| Serotype 6C | 685 | 1 | 1.77 | 0.44-6.80 | NA | NA |
| Serogroup 7 | 382 | 4 | 4.83 | 2.08-10.81 | 0.4549 | 60.7 |
| Serotype 7B | 88 | 1 | 1.14 | 0.16-7.62 | NA | NA |
| Serotype 8 | 953 | 4 | 1.01 | 0.24-4.27 | 1.5711 | 71.4 |
| Serogroup 9 | 726 | 5 | 1.82 | 1.01-3.26 | 0 | 0 |
| Serotype 9A | 257 | 3 | 1.59 | 0.60-4.15 | 0 | 0 |
| Serotype 9N | 620 | 3 | 0.68 | 0.25-1.79 | 0 | 0 |
| Serotype 9V | 389 | 3 | 2.58 | 1.35-4.89 | 0 | 0 |
| **Serogroup 10** | 326 | 3 | **6.55** | 2.73-14.89 | 0.4509 | 70 |
| **Serotype 10A** | 257 | 3 | **5.44** | 3.18-9.14 | 0 | 0 |
| Serotype 10B | 368 | 2 | 1.12 | 0.42-2.95 | 0 | 0 |
| Serotype 10C | 56 | 1 | 1.79 | 0.25-11.61 | NA | NA |
| Serotype 10F | 425 | 2 | 1.06 | 0.39-2.86 | 0.0213 | 4 |
| Serogroup 11 | 638 | 4 | 3.14 | 1.29-7.44 | 0.5357 | 64.2 |
| Serotype 11A | 168 | 2 | 3.8 | 1.71-8.20 | 0 | 0 |
| Serotype 12A | 113 | 1 | 0.88 | 0.12-6.01 | NA | NA |
| Serotype 13 | 113 | 1 | 2.65 | 0.86-7.91 | NA | NA |
| **Serotype 14** | 1312 | 9 | **5.75** | 3.97-8.36 | 0.1671 | 51.4 |
| Serogroup 15 | 456 | 3 | 0.95 | 0.36-2.52 | 0 | 0 |
| Serotype 15A | 733 | 4 | 1.27 | 0.44-3.58 | 0.5692 | 49.5 |
| Serotype 15B | 333 | 2 | 4.56 | 2.77-7.42 | 0 | 0 |
| Serotype 15C | 389 | 3 | 2.09 | 1.05-4.12 | 0 | 0 |
| Serogroup 16 | 312 | 1 | 0.32 | 0.05-2.24 | NA | NA |
| Serotype 16F | 389 | 3 | 2.13 | 0.71-6.18 | 0.4691 | 49.4 |
| Serotype 17A | 333 | 2 | 0.63 | 0.16-2.50 | 0 | 0 |
| Serotype 17F | 389 | 3 | 2.27 | 1.14-4.47 | 0 | 0 |
| Serogroup 18 | 326 | 3 | 4.25 | 2.476-7.20 | 0.002 | 0.7 |
| Serotype 18B | 312 | 1 | 0.32 | 0.05-2.24 | NA | NA |
| Serotype 18C | 214 | 3 | 2.45 | 1.02-5.57 | 0 | 0 |
| Serotype 18F | 56 | 1 | 1.79 | 0.25-11.61 | NA | NA |
| **Serogroup 19** | 119 | 3 | **25.24** | 8.07-56.48 | 1.2012 | 85.5 |
| **Serotype 19A** | 789 | 5 | **6.73** | 4.80-9.35 | 0.0466 | 28.6 |
| Serotype 19B | 364 | 3 | 0.97 | 0.31-2.97 | 0 | 0 |
| **Serotype 19F** | 834 | 6 | **7.09** | 5.52-9.07 | 0 | 0 |
| Serotype 19V | 45 | 1 | 2.22 | 0.31-14.16 | NA | NA |
| Serogroup 20 | 692 | 2 | 0.87 | 0.39-1.92 | 0 | 0 |
| Serotype 21 | 425 | 2 | 1.62 | 0.30-8.27 | 1.1298 | 74.8 |
| Serotype 22A | 645 | 3 | 1.86 | 0.99-3.46 | 0.0351 | 10.1 |
| Serotype 22F | 645 | 3 | 1.12 | 0.35-3.48 | 0.4941 | 47.2 |
| Serotype 23A | 333 | 2 | 1.94 | 0.87-4.25 | 0 | 0 |
| Serotype 23B | 389 | 3 | 4.26 | 1.31-12.97 | 0.8749 | 78.5 |
| **Serotype 23F** | 721 | 5 | **7.33** | 5.20-10.24 | 0.0467 | 27.2 |
| Serogroup 24 | 784 | 2 | 0.26 | 0.07-1.04 | 0 | 0 |
| Serotype 24B | 220 | 1 | 0.45 | 0.06-3.15 | NA | NA |
| Serotype 24F | 569 | 4 | 0.87 | 0.33-2.30 | 0 | 0 |
| Serogroup 25 | 312 | 1 | 0.64 | 0.16-2.53 | NA | NA |
| Serotype 27 | 312 | 1 | 0.64 | 0.16-2.53 | NA | NA |
| Serotype 28A | 113 | 1 | 0.88 | 0.12-6.01 | NA | NA |
| Serotype 29 | 1004 | 3 | 0.31 | 0.10-0.97 | 0 | 0 |
| Serogroup 33 | 220 | 1 | 3.18 | 1.52-6.52 | NA | NA |
| Serotype 33A | 169 | 2 | 1.26 | 0.31-4.88 | 0 | 0 |
| Serotype 33B | 169 | 2 | 2.62 | 0.45-13.69 | 1.0248 | 60.1 |
| Serotype 33C | 312 | 1 | 0.32 | 0.05-2.24 | NA | NA |
| Serotype 34 | 729 | 4 | 2.95 | 1.13-7.45 | 0.6653 | 71.9 |
| **Serogroup 35** | 220 | 1 | **6.36** | 3.80-10.46 | NA | NA |
| Serotype 35A | 456 | 3 | 2.42 | 0.73-7.69 | 0.7547 | 65.7 |
| Serotype 35B | 169 | 2 | 4.16 | 2-8.47 | 0 | 0 |
| Serotype 35C | 113 | 1 | 0.88 | 0.12-6.01 | NA | NA |
| Serotype 35F | 333 | 2 | 1.8 | 0.81-3.95 | 0 | 0 |
| Serotype 36 | 220 | 1 | 0.45 | 0.06-3.15 | NA | NA |
| Serotype 37 | 784 | 2 | 0.26 | 0.07-1.04 | 0 | 0 |
| Serotype 38 | 425 | 2 | 0.71 | 0.23-2.19 | 0 | 0 |
| Serotype 39 | 88 | 1 | 1.14 | 0.16-7.62 | NA | NA |
| Serotype 42 | 472 | 1 | 2.33 | 1.30-4.16 | NA | NA |
| Serotype 48 | 532 | 2 | 0.57 | 0.18-1.76 | 0 | 0 |
| Non Typeable | 734 | 6 | 7.66 | 3.70-15.18 | 0.6896 | 81.1 |

**Table 3:** **Serogroups/Serotypes distribution across the pediatric age group (≤2 years)**

| **Serogroups/Serotypes** | **Total No. of Isolates** | **Number of studies** | **Pooled Prevalence (%)** | **95% CI** | **Tau²** | **I²** |
| --- | --- | --- | --- | --- | --- | --- |
| **Serotype 1** | 118 | 2 | **8.01** | 4.22-14.70 | 0 | 0 |
| Serotype 2 | 472 | 1 | 0.64 | 0.21-1.95 | NA | NA |
| Serotype 3 | 500 | 2 | 0.87 | 0.05-12.68 | 3.0674 | 75.1 |
| Serotype 4 | 500 | 2 | 1.4 | 0.51-3.8 | 0.1505 | 19.5 |
| **Serotype 5** | 118 | 2 | **5.98** | 1.84-17.75 | 0.4185 | 53.8 |
| **Serogroup 6** | 28 | 1 | **28.57** | 14.98-47.59 | NA | NA |
| **Serotype 6A** | 99 | 2 | **11.66** | 4.87-25.38 | 0.1873 | 32.4 |
| **Serotype 6B** | 90 | 1 | **8.89** | 4.51-16.78 | NA | NA |
| Serotype 7F | 90 | 1 | 2.22 | 0.56 – 8.45 | NA | NA |
| Serotype 8 | 472 | 1 | 0.21 | 0.03 – 1.49 | NA | NA |
| **Serogroup 9** | 28 | 1 | **7.14** | 1.79 – 24.48 | NA | NA |
| Serotype 9V | 90 | 1 | 4.44 | 1.68 – 11.25 | NA | NA |
| Serogroup 10 | 28 | 1 | 3.57 | 0.50 – 21.42 | NA | NA |
| **Serotype 11A** | 9 | 1 | **11.11** | 1.54 – 49.99 | NA | NA |
| **Serotype 14** | 590 | 3 | **8.05** | 2.37 – 24.03 | 1.148 | 89.8 |
| Serogroup 15 | 90 | 1 | 4.44 | 1.68 – 11.25 | NA | NA |
| **Serogroup 18** | 28 | 1 | 7.14 | 1.79 – 24.48 | NA | NA |
| **Serotype 18C** | 9 | 1 | **11.11** | 1.54 – 49.99 | NA | NA |
| Serogroup 19 | 28 | 1 | **10.71** | 3.50 – 28.44 | NA | NA |
| Serotype 19A | 90 | 1 | 4.44 | 1.68 – 11.25 | NA | NA |
| **Serotype 19F** | 99 | 2 | **16.24** | 10.19 – 24.88 | 0 | 0 |
| Serogroup 20 | 472 | 1 | 0.85 | 0.32 – 2.24 | NA | NA |
| **Serotype 23F** | 9 | 1 | **22.22** | 5.60 – 57.90 | NA | NA |
| Serogroup 24 | 562 | 2 | 0.95 | 0.06 – 12.77 | 3.2118 | 82.7 |
| Serotype 29 | 472 | 1 | 0.21 | 0.03 – 1.49 | NA | NA |
| Serotype 34 | 472 | 1 | 1.48 | 0.71 – 3.08 | NA | NA |
| Serotype 37 | 472 | 1 | 0.21 | 0.03 – 1.49 | NA | NA |
| Serotype 42 | 472 | 1 | 2.33 | 1.30 – 4.16 | NA | NA |

**Figure 2: Forest plot of distribution of the common serogroups/serotypes in the pediatric age group (≤2 years)**

**Table 4: Serogroups/Serotypes distribution across the pediatric age group (3-5 years)**

| **Serogroups/Serotypes** | **Total No. of Isolates** | **Number of studies** | **Pooled Prevalence (%)** | **95% CI** | **Tau²** | **I²** |
| --- | --- | --- | --- | --- | --- | --- |
| **Serotype 1** | 48 | 1 | **8.33** | 3.16-20.19 | NA | NA |
| Serotype 4 | 42 | 1 | 2.38 | 0.33-15.06 | NA | NA |
| **Serotype 5** | 48 | 1 | **12.5** | 5.73-25.15 | NA | NA |
| **Serotype 6A** | 42 | 1 | **7.14** | 2.32-19.93 | NA | NA |
| **Serotype 6B** | 90 | 2 | **7.19** | 3.26-15.13 | 0 | 0 |
| Serotype 7F | 48 | 1 | 4.17 | 1.04 – 15.19 | NA | NA |
| Serotype 11A | 42 | 1 | 4.76 | 1.19 – 17.14 | NA | NA |
| Serotype 14 | 48 | 1 | 4.17 | 1.04 – 15.19 | NA | NA |
| **Serogroup 15** | 48 | 1 | **6.25** | 2.03 – 17.66 | NA | NA |
| **Serotype 18C** | 42 | 1 | **9.52** | 3.62 – 22.78 | NA | NA |
| **Serotype 19A** | 90 | 2 | **12.46** | 7.03 – 21.13 | 0 | 0 |
| **Serotype 19F** | 90 | 2 | **10.01** | 5.29 – 18.13 | 0 | 0 |
| **Serotype 23F** | 42 | 1 | **11.9** | 5.04 – 25.59 | NA | NA |
| Serogroup 24 | 48 | 1 | 2.08 | 0.29 – 13.36 | NA | NA |

**Figure 3: Forest plot of distribution of the common serogroups/serotypes in the pediatric age group (3-5 years)**

**Table 5: Serogroups/Serotypes distribution across the pediatric age group (5-24 years)**

| **Serogroups/Serotypes** | **Total No. of Isolates** | **Number of studies** | **Pooled Prevalence (%)** | **95% CI** | **Tau²** | **I²** |
| --- | --- | --- | --- | --- | --- | --- |
| Serotype 3 | 93 | 2 | 2.2 | 0.55-8.36 | 0 | 0 |
| **Serotype 4** | 93 | 2 | **12.26** | 4.51-29.24 | 0.393 | 63.8 |
| Serogroup 6 | 56 | 1 | 1.79 | 0.25-11.61 | NA | NA |
| **Serotype 6A** | 131 | 4 | **12.7** | 6.28-24.03 | 0.2556 | 47.5 |
| **Serotype 6B** | 113 | 3 | **5.88** | 1.95-16.42 | 0.3787 | 31.4 |
| Serogroup 7 | 56 | 1 | 1.79 | 0.25 – 11.61 | NA | NA |
| Serotype 7F | 20 | 1 | 5 | 0.70 – 28.22 | NA | NA |
| **Serotype 8** | 56 | 1 | **5.36** | 1.74 – 15.34 | NA | NA |
| Serotype 9A | 56 | 1 | 1.79 | 0.25 – 11.61 | NA | NA |
| Serotype 9V | 56 | 1 | 1.79 | 0.25 – 11.61 | NA | NA |
| Serotype 10A | 56 | 1 | 1.79 | 0.25 – 11.61 | NA | NA |
| Serotype 10B | 56 | 1 | 1.79 | 0.25 – 11.61 | NA | NA |
| Serotype 10C | 56 | 1 | 1.79 | 0.25 – 11.62 | NA | NA |
| Serotype 11A | 56 | 1 | 1.79 | 0.25 – 11.61 | NA | NA |
| **Serotype 13** | 18 | 1 | **5.56** | 0.78 – 30.65 | NA | NA |
| Serotype 14 | 76 | 2 | 4.86 | 0.87 – 22.82 | 0.8514 | 52 |
| Serogroup 15 | 70 | 2 | 4.86 | 0.87 – 22.82 | 0.8514 | 52 |
| **Serotype 15A** | 18 | 1 | **5.56** | 0.78 – 30.65 | NA | NA |
| **Serotype 15B** | 56 | 1 | **11.11** | 2.79 – 35.22 | NA | NA |
| Serotype 15C | 56 | 1 | 1.79 | 0.25 – 11.61 | NA | NA |
| **Serotype 16F** | 56 | 1 | **5.36** | 1.74 – 15.34 | NA | NA |
| Serotype 17F | 56 | 1 | 1.79 | 0.25 – 11.61 | NA | NA |
| Serotype 18C | 93 | 2 | 4.39 | 1.66 – 11.12 | 0 | 0 |
| Serotype 18F | 56 | 1 | 1.79 | 0.25 – 11.61 | NA | NA |
| **Serotype 19A** | 113 | 3 | **5.88** | 1.95 – 16.42 | 0.3787 | 31.4 |
| Serotype 19B | 56 | 1 | 1.79 | 0.25 – 11.61 | NA | NA |
| **Serotype 19F** | 131 | 4 | **9.45** | 3.98 – 20.81 | 0.4224 | 46.8 |
| **Serogroup 20** | 18 | 1 | **5.56** | 0.78 – 30.65 | NA | NA |
| **Serotype 22F** | 18 | 1 | **5.56** | 0.78 – 30.65 | NA | NA |
| Serotype 23B | 74 | 2 | 4.13 | 1.34 – 12.05 | 0 | 0 |
| Serotype 23F | 111 | 3 | 4.44 | 1.30 – 14.10 | 0.4155 | 29.4 |
| Serotype 24F | 56 | 1 | 1.79 | 0.25 – 11.61 | NA | NA |
| Serotype 33A | 56 | 1 | 1.79 | 0.25 – 11.61 | NA | NA |
| **Serotype 33B** | 56 | 1 | **5.36** | 1.74 – 15.34 | NA | NA |
| Serotype 34 | 56 | 1 | 1.79 | 0.25 – 11.61 | NA | NA |
| **Serotype 35A** | 56 | 1 | **5.36** | 1.74 – 15.34 | NA | NA |
| Serotype 35B | 56 | 1 | 3.57 | 0.89 – 13.19 | NA | NA |
| **Serotype 38** | 18 | 1 | **5.56** | 0.78 – 30.65 | NA | NA |
| Non Typeable | 56 | 1 | 10.71 | 4.89 – 21.87 | NA | NA |

**Figure 4: Forest plot of distribution of the common serogroups/serotypes in the pediatric age group (5-24 years)**

**Table 6: Serogroups/Serotypes distribution across the pediatric age group (≤5 years)**

| **Serogroups/Serotypes** | **Total No. of Isolates** | **Number of studies** | **Pooled Prevalence (%)** | **95% CI** | **Tau²** | **I²** |
| --- | --- | --- | --- | --- | --- | --- |
| **Serotype 1** | 1045 | 11 | **7.71** | 5.34-11.01 | 0.1718 | 51.3 |
| Serotype 2 | 560 | 3 | 1.61 | 0.55-4.59 | 0.341 | 43.2 |
| Serotype 3 | 1376 | 9 | 1.96 | 0.90-4.23 | 0.9054 | 69.7 |
| Serotype 4 | 926 | 7 | 1.85 | 0.82-4.12 | 0.5689 | 46.5 |
| **Serotype 5** | 1264 | 9 | **5.88** | 3.56-9.57 | 0.4252 | 69.8 |
| Serogroup 6 | 519 | 5 | 2.97 | 0.93-9.10 | 1.1659 | 62.4 |
| **Serotype 6A** | 1648 | 12 | **7.17** | 5.38-9.50 | 0.1342 | 51.4 |
| **Serotype 6B** | 1914 | 15 | **8.47** | 5.76-12.29 | 0.4758 | 76 |
| Serotype 6C | 1210 | 8 | 1.09 | 0.61 – 1.97 | 0 | 0 |
| Serotype 6D | 185 | 1 | 1.08 | 0.27 – 4.22 | NA | NA |
| **Serogroup 7** | 106 | 2 | **8.9** | 4.59 – 16.54 | 0.0144 | 5 |
| Serotype 7A | 242 | 2 | 0.9 | 0.23 – 3.54 | 0 | 0 |
| Serotype 7B | 449 | 2 | 0.56 | 0.14 – 2.22 | 0.0013 | 0.1 |
| Serotype 7C | 361 | 1 | 1.39 | 0.58 – 3.28 | NA | NA |
| Serotype 7F | 655 | 4 | 2.33 | 0.70 – 7.50 | 1.1193 | 80.4 |
| Serotype 8 | 1178 | 6 | 0.65 | 0.31 – 1.35 | 0 | 0 |
| Serogroup 9 | 506 | 4 | 1.52 | 0.60 – 3.77 | 0.208 | 9.6 |
| Serotype 9A | 747 | 4 | 1.01 | 0.48 – 2.11 | 0 | 0 |
| Serotype 9N | 839 | 5 | 1.2 | 0.56 – 2.55 | 0.1589 | 0 |
| Serotype 9V | 1419 | 12 | 4.96 | 3.55 – 6.90 | 0.1174 | 37.9 |
| **Serogroup 10** | 106 | 2 | **9.56** | 4.16 – 20.48 | 0.1741 | 40 |
| Serotype 10A | 1296 | 10 | 3.05 | 1.84 – 5.03 | 0.3156 | 48.1 |
| Serotype 10B | 312 | 1 | 0.96 | 0.31 – 2.94 | NA | NA |
| Serotype 10C | 278 | 3 | 1.31 | 0.42 – 3.99 | 0 | 0 |
| Serotype 10F | 1178 | 6 | 1.32 | 0.78 – 2.21 | 0 | 0 |
| Serogroup 11 | 418 | 3 | 2.8 | 0.65 – 11.30 | 1.2988 | 76.1 |
| Serotype 11A | 1318 | 9 | 3.1 | 2.02 – 4.74 | 0.1277 | 38.9 |
| Serotype 11C | 72 | 1 | 1.39 | 0.20 – 9.20 | NA | NA |
| Serotype 11D | 293 | 2 | 0.71 | 0.18 – 2.78 | 0 | 0 |
| Serotype 11F | 185 | 1 | 0.54 | 0.08 – 3.73 | NA | NA |
| Serotype 12A | 298 | 2 | 1.01 | 0.33 – 3.09 | 0 | 0 |
| Serotype 12F | 531 | 2 | 0.68 | 0.17 – 2.66 | 0.3046 | 28.8 |
| Serotype 13 | 623 | 4 | 1.65 | 0.86 – 3.15 | 0 | 0 |
| **Serotype 14** | 2008 | 18 | **10.88** | 8.22 – 14.27 | 0.2829 | 71.9 |
| Serogroup 15 | 400 | 2 | 0.78 | 0.25 – 2.38 | 0 | 0 |
| Serotype 15A | 1398 | 8 | 1.85 | 0.93 – 3.67 | 0.5111 | 57.2 |
| Serotype 15B | 1318 | 9 | 2.58 | 1.75 – 3.78 | 0.0298 | 16.1 |
| Serotype 15C | 1138 | 7 | 2.73 | 1.12 – 6.52 | 1.071 | 78.6 |
| Serotype 15F | 546 | 2 | 0.66 | 0.18 – 2.35 | 0.184 | 19.6 |
| Serogroup 16 | 312 | 1 | 0.32 | 0.05 – 2.24 | NA | NA |
| Serotype 16F | 984 | 5 | 0.86 | 0.43 – 1.72 | 0 | 0 |
| Serogroup 17 | 108 | 1 | 0.93 | 0.13 – 6.28 | NA | NA |
| Serotype 17A | 113 | 1 | 0.88 | 0.12 – 6.01 | NA | NA |
| Serotype 17F | 1138 | 7 | 1.68 | 0.85 – 3.31 | 0.316 | 36 |
| **Serogroup 18** | 106 | 2 | **5.97** | 2.70 – 12.68 | 0 | 0 |
| Serotype 18A | 851 | 5 | 1.31 | 0.49 – 3.50 | 0.5834 | 46.3 |
| Serotype 18B | 673 | 2 | 0.3 | 0.07 – 1.18 | 0 | 0 |
| Serotype 18C | 1189 | 9 | 2.62 | 1.49 – 4.56 | 0.3631 | 48.7 |
| Serotype 18F | 505 | 3 | 1.26 | 0.48 – 3.27 | 0.1261 | 12.9 |
| **Serogroup 19** | 119 | 3 | **25.4** | 6.93 – 60.88 | 1.587 | 85.5 |
| **Serotype 19A** | 1919 | 13 | **5.77** | 4.49 – 7.39 | 0.0771 | 38.5 |
| Serotype 19B | 747 | 5 | 1.12 | 0.53 – 2.34 | 0.0001 | 3.8 |
| **Serotype 19F** | 2002 | 16 | **9.61** | 8.01 – 11.49 | 0.0494 | 36.4 |
| Serotype 19V | 45 | 1 | 2.22 | 0.31 – 14.16 | NA | NA |
| Serogroup 20 | 618 | 4 | 1.4 | 0.53 – 3.69 | 0.4287 | 39.7 |
| Serotype 21 | 800 | 4 | 1.75 | 0.62 – 4.88 | 0.6881 | 61.3 |
| Serotype 22A | 934 | 6 | 1.71 | 1.01 – 2.86 | 0 | 0 |
| Serotype 22F | 1306 | 7 | 1.49 | 0.91 – 2.42 | 0 | 0 |
| Serotype 23A | 1170 | 7 | 0.82 | 0.41 – 1.64 | 0 | 0 |
| Serotype 23B | 453 | 3 | 2.71 | 0.59 – 11.50 | 1.5001 | 85.4 |
| **Serotype 23F** | 1707 | 11 | **6.69** | 5.39 – 8.28 | 0.0317 | 28.8 |
| Serogroup 24 | 312 | 1 | 0.32 | 0.05 – 2.24 | NA | NA |
| Serotype 24A | 361 | 1 | 0.28 | 0.04 – 1.94 | NA | NA |
| Serotype 24B | 541 | 4 | 0.98 | 0.41 – 2.34 | 0 | 0 |
| Serotype 24F | 1198 | 7 | 0.85 | 0.44 – 1.63 | 0 | 0 |
| Serogroup 25 | 312 | 1 | 0.64 | 0.16 – 2.53 | NA | NA |
| Serotype 25A | 701 | 3 | 0.46 | 0.15 – 1.41 | 0 | 0 |
| Serotype 27 | 1069 | 6 | 1.16 | 0.50 – 2.69 | 0.4234 | 36.5 |
| Serotype 28A | 113 | 1 | 0.88 | 0.12 – 6.01 | NA | NA |
| Serotype 28F | 546 | 2 | 0.66 | 0.18 – 2.35 | 0.184 | 19.6 |
| Serotype 29 | 1182 | 6 | 0.63 | 0.28 – 1.39 | 0 | 0 |
| Serotype 31 | 361 | 1 | 0.28 | 0.04 – 1.94 | NA | NA |
| Serogroup 33 | 531 | 2 | 0.57 | 0.18 – 1.74 | 0 | 0 |
| Serotype 33A | 659 | 3 | 0.51 | 0.16 – 1.57 | 0 | 0 |
| Serotype 33B | 659 | 3 | 0.93 | 0.33 – 2.59 | 0.1931 | 16.1 |
| Serotype 33C | 636 | 4 | 1.22 | 0.55 – 2.70 | 0.0001 | 0 |
| Serotype 33D | 185 | 1 | 0.54 | 0.08 – 3.73 | NA | NA |
| Serotype 33F | 577 | 4 | 1.59 | 0.83 – 3.04 | 0 | 0 |
| Serotype 34 | 1072 | 6 | 2.66 | 1.20 – 5.78 | 0.6978 | 68.4 |
| Serotype 35A | 1101 | 5 | 1.65 | 0.85 – 3.17 | 0.2334 | 41.9 |
| Serotype 35B | 814 | 4 | 1.15 | 0.34 – 3.81 | 0.9619 | 68.3 |
| Serotype 35C | 453 | 3 | 4.2 | 2.44 – 7.14 | 0.0459 | 43.7 |
| Serotype 35F | 828 | 5 | 1.84 | 0.52 – 6.33 | 1.6024 | 79.8 |
| Serotype 36 | 546 | 2 | 1.01 | 0.25 – 3.94 | 0 | 0 |
| Serotype 37 | 312 | 1 | 0.32 | 0.05 – 2.24 | NA | NA |
| Serotype 38 | 1213 | 6 | 0.93 | 0.50 – 1.73 | 0 | 0 |
| Serotype 39 | 712 | 5 | 0.9 | 0.40 – 1.99 | 0 | 0 |
| Serotype 40 | 361 | 1 | 1.89 | 0.27 – 12.21 | NA | NA |
| Serotype 41F | 361 | 1 | 1.89 | 0.27 – 12.21 | NA | NA |
| Serotype 42 | 185 | 1 | 0.54 | 0.08 – 3.73 | NA | NA |
| Serotype 45 | 546 | 2 | 1.01 | 0.25 – 3.94 | 0 | 0 |
| Serotype 47F | 284 | 2 | 1.22 | 0.39 – 3.71 | 0 | 0 |
| Serotype 48 | 673 | 2 | 0.6 | 0.22 – 1.58 | 0 | 0 |
| Non Typeable | 891 | 6 | 4.02 | 1.46 – 10.60 | 1.2947 | 87 |

**Figure 5: Forest plot of distribution of the common serogroups/serotypes in the pediatric age group (≤5 years)**

**Table 7:** **Serogroups/Serotypes distribution across adult population – IPD**

| **Serotypes** | **Total No. of Isolates** | **Number of studies** | **Pooled Prevalence (%)** | **95% CI** | **Tau²** | **I²** |
| --- | --- | --- | --- | --- | --- | --- |
| **Serotype 1** | 906 | 3 | **11.69** | 5.18–24.29 | 0.4552 | 92.5% |
| Serotype 2 | 402 | 1 | 0.50 | 0.12-1.97 | NA | NA |
| **Serotype 3** | 906 | 3 | **7.00** | 4.89–9.94 | 0.0467 | 42.3% |
| Serotype 4 | 490 | 2 | 3.30 | 2.03-5.32 | 0 | 0 |
| **Serotype 5** | 402 | 1 | **5.97** | 4.03-8.75 | NA | NA |
| **Serotype 6A** | 490 | 2 | **5.39** | 1.16–21.71 | 1.1957 | 91.7% |
| Serotype 6B | 490 | 2 | 3.31 | 2.04–5.34 | 0 | 0.00% |
| Serotype 6C | 402 | 1 | 1.24 | 0.52-2.95 | NA | NA |
| Serotype 7A | 402 | 1 | 0.25 | 0.04-1.74 | NA | NA |
| Serotype 7B | 490 | 2 | 2.34 | 0.65-8.10 | 0.652 | 73.7 |
| Serotype 7C | 402 | 1 | 0.25 | 0.01-1.38 | NA | NA |
| Serotype 7F | 490 | 2 | 2.66 | 1.55-4.53 | 0 | 0 |
| **Serotype 8** | 490 | 2 | **5.24** | 2.37–11.18 | 0.2868 | 57.2% |
| Serotype 9B | 402 | 1 | 0.25 | 0.04–1.74 | NA | NA |
| **Serotype 9N** | 402 | 2 | **5.15** | 1.68–14.68 | 0.5829 | 68% |
| **Serotype 9V** | 490 | 2 | **5.15** | 1.68–14.68 | 0.5829 | 83.9% |
| Serotype 10A | 402 | 1 | 0.5 | 0.12–1.97 | NA | NA |
| Serotype 10B | 402 | 1 | 0.25 | 0.04–1.74 | NA | NA |
| Serotype 10C | 402 | 1 | 0.75 | 0.24–2.29 | NA | NA |
| Serotype 10F | 402 | 1 | 0.25 | 0.04–1.74 | NA | NA |
| Serotype 11A | 402 | 1 | 0.75 | 0.15–2.17 | NA | NA |
| Serotype 12B | 402 | 1 | 1 | 0.37–2.62 | NA | NA |
| Serotype 12F | 402 | 1 | 1.49 | 0.67–3.28 | NA | NA |
| Serotype 13 | 490 | 2 | 2.67 | 1.56-4.55 | NA | NA |
| Serotype 14 | 490 | 2 | 4.82 | 3.16-7.28 | 0.0074 | 5.80% |
| Serotype 15A | 402 | 1 | 2.24 | 1.17-4.25 | NA | NA |
| Serotype 15B | 402 | 1 | 0.25 | 0.04-1.74 | NA | NA |
| Serotype 15F | 402 | 1 | 1 | 0.37-2.62 | NA | NA |
| Serotype 16F | 402 | 1 | 1 | 0.37-2.62 | NA | NA |
| Serotype 17F | 490 | 2 | 1.21 | 0.41-3.53 | 0.2138 | 33.5% |
| Serotype 18A | 490 | 2 | 0.65 | 0.21-2.01 | 0 | 0 |
| Serotype 18B | 402 | 1 | 0.25 | 0.04-1.74 | NA | NA |
| Serotype 18C | 402 | 1 | 0.5 | 0.12-1.97 | NA | NA |
| Serotype 18F | 402 | 1 | 0.75 | 0.24-2.29 | NA | NA |
| Serotype 19A | 490 | 2 | 3.69 | 2.34-5.79 | 0 | 0 |
| Serotype 19B | 402 | 1 | 0.50 | 0.12-1.97 | NA | NA |
| Serotype 19C | 402 | 1 | 0.50 | 0.12-1.97 | NA | NA |
| **Serotype 19F** | 490 | 2 | **6.71** | 4.61-9.66 | 0.0123 | 11.9% |
| Serogroup 20 | 402 | 1 | 0.5 | 0.12-1.97 | NA | NA |
| Serotype 21 | 402 | 1 | 0.5 | 0.12-1.97 | NA | NA |
| Serotype 22A | 402 | 1 | 1 | 0.37-2.62 | NA | NA |
| Serotype 23A | 402 | 1 | 1.74 | 0.83-3.61 | NA | NA |
| Serotype 23B | 402 | 1 | 0.75 | 0.24-2.29 | NA | NA |
| **Serotype 23F** | 490 | 2 | **6.84** | 2.52-17.25 | 0.481 | 84.7% |
| Serogroup 24 | 402 | 1 | 0.25 | 0.04-1.74 | NA | NA |
| Serogroup 25F | 402 | 1 | 1 | 0.37-2.62 | NA | NA |
| Serotype 28A | 402 | 1 | 0.25 | 0.04-1.74 | NA | NA |
| Serotype 28F | 402 | 1 | 0.25 | 0.04-1.74 | NA | NA |
| Serotype 29 | 402 | 1 | 0.25 | 0.04-1.74 | NA | NA |
| Serotype 31 | 402 | 1 | 1.49 | 0.67-3.28 | NA | NA |
| Serotype 33B | 402 | 1 | 0.25 | 0.04-1.74 | NA | NA |
| Serotype 33C | 402 | 1 | 0.25 | 0.04-1.74 | NA | NA |
| Serotype 33F | 402 | 1 | 0.75 | 0.24-2.29 | NA | NA |
| Serotype 34 | 402 | 1 | 0.5 | 0.12-1.97 | NA | NA |
| Serotype 35A | 402 | 1 | 0.25 | 0.04-1.74 | NA | NA |
| Serotype 35B | 402 | 1 | 0.75 | 0.24-2.29 | NA | NA |
| Serotype 35F | 402 | 1 | 0.25 | 0.04-1.74 | NA | NA |
| Serotype 36 | 402 | 1 | 0.25 | 0.04-1.74 | NA | NA |
| Serotype 37 | 402 | 1 | 0.25 | 0.04-1.74 | NA | NA |
| Serotype 38 | 490 | 2 | 3.98 | 0.99-14.64 | 0.9075 | 86.5% |
| Serotype 39 | 402 | 1 | 1 | 0.37-2.62 | NA | NA |
| Serotype 45 | 490 | 2 | 1.21 | 0.41-3.53 | 0.2138 | 33.5% |
| Serotype 48 | 402 | 1 | 0.25 | 0.04-1.74 | NA | NA |
| Non Typeable | 402 | 1 | 1.24 | 0.52-2.95 | NA | NA |

**Table 8:** **Serogroups/Serotypes distribution across adult population – CAP**

| **Serogroup/Serotype** | **Total No. of Isolates** | **Number of studies** | **Pooled Prevalence (%)** | **95% CI** | **Tau²** | **I²** |
| --- | --- | --- | --- | --- | --- | --- |
| Serotype 1 | 50 | 1 | 4.00 | 1.0–14.63 | NA | NA |
| **Serotype 3** | 50 | 1 | **6.00** | 1.95–17.02 | NA | NA |
| Serotype 5 | 50 | 1 | 2.00 | 0.28-12.88 | NA | NA |
| Serotype 6A | 50 | 1 | 4.00 | 1.00–14.63 | NA | NA |
| Serotype 7F | 50 | 1 | 2.00 | 0.28-12.88 | NA | NA |
| **Serotype 8** | 50 | 1 | **10.00** | 4.22-21.87 | NA | NA |
| Serotype 9N | 50 | 1 | 4.00 | 1.00–14.63 | NA | NA |
| Serotype 9V | 50 | 1 | 4.00 | 1.00–14.63 | NA | NA |
| Serotype 11A | 50 | 1 | 2.00 | 0.28-12.88 | NA | NA |
| Serotype 17F | 50 | 1 | 2.00 | 0.28-12.88 | NA | NA |
| **Serotype 19A** | 50 | 1 | **14.00** | 6.82-26.57 | NA | NA |
| **Serotype 19F** | 50 | 1 | **8.00** | 3.04-19.46 | NA | NA |
| Serogroup 20 | 50 | 1 | 2.00 | 0.28-12.88 | NA | NA |
| Serotype 23F | 50 | 1 | 4.00 | 1.00-14.63 | NA | NA |
| NVT | 50 | 1 | 30 | 18.97-43.97 | NA | NA |

**Figure 6: PCV serotype coverage (IPD) approved for the pediatric population**

| Vaccines | Coverage (%) |
| --- | --- |
| PCV10-Protein D, TT, & DT (including 4, 18C) | 66 |
| PCV10-CRM197 (including 6A, 19A) | 73.86 |
| PCV 13 | 77.67 |
| PCV 14 | 73.78 |
| PCV 15 (including 2, 12F) | 78.41 |
| PCV 15 (including 22F, 33F) | 78.83 |
| PCV 20 | 83.04 |

**Figure 7: PCV serotype coverage (Carriage) approved for the pediatric population**

| Vaccines | Coverage (%) |
| --- | --- |
| PCV10-Protein D, TT, & DT (including 4, 18C) | 32.21 |
| PCV10-CRM197 (including 6A, 19A) | 40.67 |
| PCV 13 | 44.9 |
| PCV 14 | 40.8 |
| PCV 15 (including 2, 12F) | 45.27 |
| PCV 15 (including 22F, 33F) | 45.65 |
| PCV 20 | 50.75 |

**Figure 8: Pneumococcal vaccine serotype coverage (IPD) across vaccine serotypes approved for the adult population**

| Vaccines | Coverage (%) |
| --- | --- |
| PCV 13 | 69.97 |
| PCV 15 (including 22F, 33F) | 70.47 |
| PCV 20 | 76.01 |
| PCV 21 | 31.88 |
| PPSV 23 | 74.66 |

**Figure 9: Pneumococcal vaccine serotype coverage (CAP) approved for the adult population**

| Vaccines | Coverage (%) |
| --- | --- |
| PCV 13 | 48 |
| PCV 15 (including 22F, 33F) | 48 |
| PCV 20 | 60 |
| PCV 21 | 46 |
| PPSV 23 | 66 |
